# Quantitation and Identification of Intact Major Milk Proteins for High-Throughput LC-ESI-Q-TOF MS Analyses

**DOI:** 10.1371/journal.pone.0163471

**Published:** 2016-10-17

**Authors:** Delphine Vincent, Aaron Elkins, Mark R. Condina, Vilnis Ezernieks, Simone Rochfort

**Affiliations:** 1 Department of Economic Development, Jobs, Transport and Resources, AgriBio Centre, 5 Ring Road, Bundoora, Victoria 3083, Australia; 2 Bruker Pty. Ltd, Preston, Victoria, Australia; 3 La Trobe University, Bundoora, Victoria 3083, Australia; Università degli Studi di Milano, ITALY

## Abstract

Cow’s milk is an important source of proteins in human nutrition. On average, cow’s milk contains 3.5% protein. The most abundant proteins in bovine milk are caseins and some of the whey proteins, namely beta-lactoglobulin, alpha-lactalbumin, and serum albumin. A number of allelic variants and post-translationally modified forms of these proteins have been identified. Their occurrence varies with breed, individuality, stage of lactation, and health and nutritional status of the animal. It is therefore essential to have reliable methods of detection and quantitation of these proteins. Traditionally, major milk proteins are quantified using liquid chromatography (LC) and ultra violet detection method. However, as these protein variants co-elute to some degree, another dimension of separation is beneficial to accurately measure their amounts. Mass spectrometry (MS) offers such a tool. In this study, we tested several RP-HPLC and MS parameters to optimise the analysis of intact bovine proteins from milk. From our tests, we developed an optimum method that includes a 20-28-40% phase B gradient with 0.02% TFA in both mobile phases, at 0.2 mL/min flow rate, using 75°C for the C8 column temperature, scanning every 3 sec over a 600–3000 m/z window. The optimisations were performed using external standards commercially purchased for which ionisation efficiency, linearity of calibration, LOD, LOQ, sensitivity, selectivity, precision, reproducibility, and mass accuracy were demonstrated. From the MS analysis, we can use extracted ion chromatograms (EICs) of specific ion series of known proteins and integrate peaks at defined retention time (RT) window for quantitation purposes. This optimum quantitative method was successfully applied to two bulk milk samples from different breeds, Holstein-Friesian and Jersey, to assess differences in protein variant levels.

## 1. Introduction

Bovine milk has been consumed by humans for as long as 8000 years in some regions of the globe; now human consumption of cow milk is world-wide, crosses all age groups, but is particularly prevalent during childhood as a result in part of promotional marketing especially in Asia where drinking milk is not part of the culture[1]. Becauseof bovine milk’s nutritional and economical values, dairycattle breeds have been efficiently selected and successfully bred for increased milk production for centuries[2]. Through the combined effects of breeding, improved nutrition and husbandry practices, milk production of the modern dairy cow now far exceeds offspring requirements [3]. This milk excess is then offered on commercial marketsfor human nutrition as fresh pasteurised liquid milk, or further processed into yogurt, butter, cream, cheese, cream cheese, ice cream, powdered milk *etc…* to name a few of the mainstream dairy products. Breed is recognised as one of the main factors affecting milk composition and properties. Cattle breeds of the species *Bostaurus*, produce 85% of all milk commercially sold[2]; examples of these main breeds includeHolstein and Jersey. A nation-wide study comprising 90.1% Holstein and 5.3% Jersey of the 2009 United States dairy herd revealed that on average Holstein and Jersey cows daily produced 29.1 and 20.9 kg of milk, respectively with an average protein content of 3.1 and 3.7% [4]. In a different study, it was reported that although Jersey milk had greater gross value than Holstein’s due to higher protein content, total volume of milk produced by Holstein cows offset this difference [5].

On average, cow’s milk contains about 3.5% protein;however this level can vary with breed, individuality, stage of lactation, and health and nutritional status of the animal. The functional properties of milk proteins have been reviewed [6]. Caseins represent about 80% of total bovine milk proteins and whey proteins about 18%[2]. There arefive different types of caseins: alpha-S1-casein (aS1CN), alpha-S2-casein (aS2CN), beta-casein (bCN), kappa-casein (kCN), and gamma-casein (gCN)the latter being breakdown products cleaved from bCN by the major milk proteolytic enzyme plasmin[3]. The aS1-, aS2-, b-, and k-caseins are on average found at the following proportions in cow’s milk, 38, 10, 35, and 12%, respectively. Caseins are of relatively small molecular weight (20–25 kDa). The four most abundant whey proteins are beta-lactoglobulin (bLG), alpha-lactalbumin (aLA), bovine serum albumin (BSA), and immunoglobulins (Igs), which represent approximately 60, 20, 10, and 10% of total whey proteins, respectively. BSA is a leakage protein from blood which bears no biological or technological significance in milk [2]. These major milk proteins are encoded by highly polymorphic genes for which non synonymous and synonymous mutations have been reported, thus giving rise to 53 naturally occurring protein variants. The list, features and sequence information of all variants for aS1CN, aS2CN, bCN, kCN, aLA and bLG proteins has been summarised[7], and further updated [8–10]. There are currently 9 aS1CN variants, 4 aS2CN variants, 13 bCN variants, 13 kCN variants, 3 aLA variants and 11 bLG variants that have been described. These genetic variations mainly result in AA exchanges or deletions within the coding sequences thereby impacting the function of the encoded protein. Mutations within the noncoding sequences have been shown to affect protein expression and, in turn, milk composition which bears consequences on subsequent manufacturing steps, for example cheese making. The study of milk protein variants can be applied to breed characterization, diversity, and phylogeny. Furthermore, because milk proteins are involved in various aspects of human diet, characterising the occurrence of alleles associated with a reduced content of different caseins might be exploited for the production of hypoallergenic milk[8]. Beside allelic variations, major milk proteins are heavily post-translationally modified with varying levels of phosphorylation of serine or threonine and/or gylcosylation of threonine residues, proteolysis by the indigenous milk enzymes, and oxidation of cysteine to disulfide bonds [9]. The number of phosphorylated groups (P) attached to caseins is variable, from 1P to 3P on kCNs, 4P to 5P on bCNs, 8P to 9P on aS1CN, and 10P to 13P on aS2CN [7,10–11]. Through these phosphorylation sites, caseins bond to the hydrated calcium phosphate entities present in the casein micelles, thus stabilising their structure[9]. About half of the kCNs are glycosylated with short oligosaccharide chains at one or several threonine sites, and most of the kCNs are phosphorylated at Ser149 [9]; casein micelle size has been correlated with the presence of glycosylation on kCN[12].

The fractionation and isolation of intact milk proteins for their subsequent analysis depend on the intrinsic physicochemical properties of the individual proteins. Owing to the aggregating nature of proteins, a denaturing reaction is required prior to separation. Chaotropes and reducing reagents are commonly employed; for instance, the denaturant guanidine hydrochloride in combination with the reductant dithiothreitol (DTT) have often been used [13–17]. Alternatively, urea combined with mercaptoethanolhas also been frequently employed [18–22]. Among the diverse chromatographic and electrophoretic fractionation strategies that exist [10, 23 for review], liquid chromatography (LC) remains the most commonly employed for analytical purposes, and in particular reversed-phase high performance liquid chromatography (RP-HPLC) which separates compounds based on their hydrophobicity. The stationary phase of RP-HPLC separation columns is nonpolar and typically made of silanized silica with C4, C8 or C18 groups coupled to the silanol groups [23]. For instance, C18 columns [13, 22, 24], C8 columns [15, 21], and C4 columns [14, 16– 18, 25] have all been employed for milk protein analysis. More recently, a C4 HPLC column was compared to a monolithic capillary HPLC column, with the latter displaying a greater resolving power [19]. Bobe *et al* [13] introduced a standard protocol for intact milk proteins separation by gradient elution at low pH with 0.1% trifluoroacetic acid (TFA) added to the mobile phases, thus avoiding aggregation and non-specific interactions of milk proteins and improving both protein solubilisation and chromatographic resolution. This 0.1% TFA concentration has since often been employed to study intact milk proteins[14–19, 22, 24–25]. Whilst at low concentrations, TFA helps recover larger proteins by enhancing their solubilisation; at high concentrations (≥0.1%), TFA is known to suppress ionization of analytes in the electrospray ionisation (ESI) source of the mass spectrometer. Therefore, in the afore-mentioned studies, the proteins were only detected and quantified online by measuring ultraviolet (UV) absorbance at 210–220 nm, and not using a mass spectrometer. If chromatographic separation is compatible with MS, then the analysis of proteins using a mass spectrometer adds another orthogonal separation dimension to the LC, further separating proteins by their mass which not only improves the selectivity of the analysis but also gives access to protein identities. Details of the published masses of bovine milk proteins obtained using MS can be found in the Supplementary information (S1 File).

**Fig 1:**
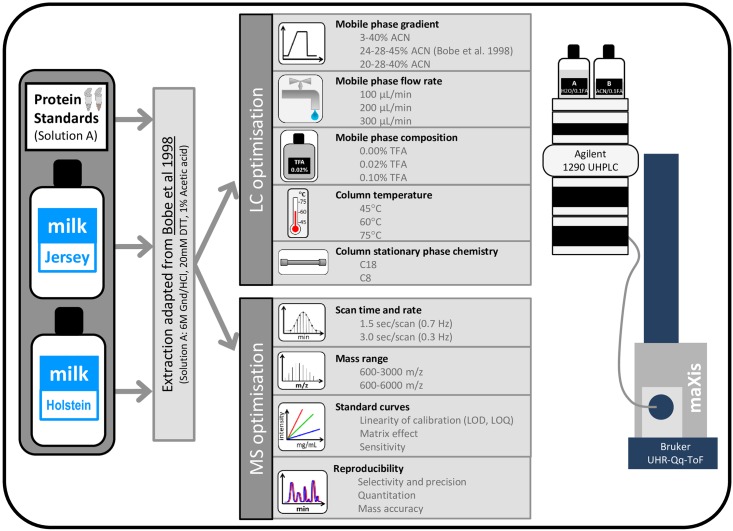
Experimental design.

The aim of the present study was not to optimise the preparation of milk samples for intact protein analysis as it has been well established [13–17, 24–25]. Rather, this works aims at optimising HPLC separation and MS analysis to identify and quantify cow milk proteins in a high-throughput manner. Fig 1 outlines the experimental design of the study. We have first optimised HPLC and MS settings using milk protein external and internal standards by assessing the linearity of calibration, matrix effect, sensitivity, reproducibility, selectivity, precision and mass accuracy. We also compared UV chromatograms and Base Peak Chromatograms (BPCs) to Extracted Ion Chromatograms (EICs). We then applied our optimum parameters to bulk milk samples from two bovine breeds, Hosltein-Friesian and Jersey, to validate the quantitative method.

## 2. Materials and Methods

Fig 1 summarises the HPLC and MS parameters that were tested for method validation.

### 2.1. Skim milk sample preparation

Milk sampling and skimming has been described [26]. The pasture-fed Holstein-Friesian and Jersey cows (Gippsland region, Victoria, Australia) were cared for in accordance with the Australian Code of Practice for the Care and Use of Animals for Scientific Purposes (www.nhmrc.gov.au). The experiment received animal ethics approval from the Agricultural Research and Extension Animal Ethics Committee of the Department of Economic Development, Jobs, Transport and Resources (Victoria, Australia). No particular steps were needed to ameliorate pain and suffering of the animals because cows were not subjected to any pain inducing procedures. Cows were exposed to the same type of handling, management and milk sampling that occurs on Australian commercial dairy farms. Proportional samplers (DeLaval International, Tumba, Sweden) were used to collect a sample of milk from each cow at each milking. Cows were milked twice daily, at 6:00 and 15:00, and milk was bulked into containers. A 50 mL aliquot of bulk milk samples from Jersey cows and from Holstein-Friesian cows were separately collected on 6 November 2014 and stored on ice at the respective dairy farms and during transport. A total of 440 Holstein-Friesian cows contributed to the vat on that date and cows averaged 139 days in milk. A total of 215 Jersey cows contributed to the vat on that date and cows averaged 140 days in milk. Three 2.0 mL milk samples were aliquoted from each bulk sample and stored at -80C until use. Milk protein extracts were prepared following method from [13] with modifications. A 0.5 mL volume of cold skim milk was transferred into a 1.5mL tube and 0.5 mL of Solution A (0.1 M Bis-Tris, 6 M Gdn-HCl, 5.37 mM sodium citrate tribasic dehydrate, and 20 mM DTT) was added. The mixture was vortexed for 1 min and left to incubate at room temperature for 50 min. A 0.02 mL volume of 50% acetic acid (1% acetic acid final concentration, pH 5.8) was then added to the milk/Solution A mixture. The tube was vortexed for 1 min and left to incubate at room temperature for 10 min. A 0.1 mL aliquot of the milk protein extract was transferred into a 100μL glass insert placed into a 2mL glass vial for immediate analysis.

### 2.2. Bovine external standard preparation and internal standard

In order to optimise HPLC separation, bovine protein standards were purchased from Sigma. The protein standards include: α-casein (aCN) from bovine milk (C6780-250MG, 70% pure), β-casein (bCN) from bovine milk (C6905-250MG, 98% pure), κ-casein (kCN) from bovine milk (C0406-250MG, 70% pure), α-lactalbumin(aLA) from bovine milk (L5385-25MG, 85% pure), β-lactoglobulin(bLG) from bovine milk (L3908-250MG, contains lactoglobulins A and B, 90% pure), albumin from bovine serum (BSA, A7906-10G, 98% pure). These lyophilised protein standards were fully solubilised at a 10mg/mL concentration in 50% solution 1/50% MilliQ H_2_O. Standards were dissolved by vortexing for 1 min and sonication for 5 min followed by another 1 min vortexing. Solubilised standards were left for 50 min at room temperature. A volume of 50% acetic acid to reach 1% acetic acid final concentration was added to the standards. Care was taken not to lower the pH below 4.6 as it would precipitate caseins; under our conditions pH was 5.5. Standards were vortexed for 1 min and left to incubate at room temperature for 10 min. A 0.1 mL aliquot of the solubilised standard was transferred into a 100 μL glass insert placed into a 2 mL glass vial for immediate analysis.

Myoglobin (Myo) from horse skeletal muscle was purchased from Sigma (M0630-250MG, 95–100% pure, essentially salt-free) and spiked as an internal standard (IS). A 10mg/mL myoglobin solution was prepared as described above. A 98μL milk protein extract was spiked with 2μL myoglobin solution (0.2mg/mL Myoglobin final concentration).

### 2.3. HPLC separation

Prior to analysis by MS, bovine milk proteins and standards were chromatographically separated using the UHPLC 1290 Infinity Binary LC system (Agilent). For method optimisation purpose, a series of parameters were modified as described in Fig 1 and detailed in the Supplementary information (S1 File).

The settings common to all tests are listed hereafter. The injection volume was 3μL (with needle wash). The diode array detector (DAD) spectrum was acquired from 190 to 400 nm. The pressure limit was set at 600 bars. The total duration of the HPLC separation was 40 min, with the first 2.5 min switched to waste to allow for online desalting and infusion of the internal calibrant (Na formate solution composed of 1M NaOH in 50% isopropanol (IPA)/0.1% formic acid (FA)) into the mass spectrometer.

### 2.4. MS analysis

HPLC and MS parameters were set using microToF 3.4, ESI Compass 1.3 andHyStarPP 3.2SR4 software (Bruker DaltonikGmbh). Following HPLC separation, milk proteins were analysed using a maXis HD UHR-Qq-ToF (60,000 resolution) with an ESI source (BrukerDaltonikGmbh). The MS was calibrated weekly and auto-tuned monthly using the ESI-L low concentration tuning mix (Agilent).

To ensure mass accuracy, a Na-formate solution was infused continuously at a 0.1 mL/h and the first 2.5 min of each run were used to re-calibrate masses post-acquisition. Each 40 min run was thus segmented as follows: 2.5 min to waste and the following 37.5 min to source. Capillary voltage was set at 4500V. The nebulizer was set at 1.5 bar. The dry gas was set at 8 L/min. The dry temperature was set at 190°C. The transfer funnel RF and multipole RF were set at 400Vpp, no ISCID energy was applied. The quadrupole ion energy was 5eV, the collision cell energy was 10eV and the collision RF 1800Vpp. The ion cooler transfer time was 120 μs, with a prepulse storage of 10 μs, and a RF of 400Vpp. The ion polarity was positive and scan mode was MS. The rolling average mode was activated and set at 2.

Details of the MS parameters tested and mass spectra deconvolution can be found in the Supplementary information (S1 File). Extracted ion chromatograms (EICs) were produced for each standard using the ion series indicated in Table A in S2 File and a +/- 0.1 m/z tolerance. For a given standard and a given dilution, the peak areas of each individual protein variant were summed as a proxy for the standard response. Peak areas were integrated using the retention times (RT) indicated in Table A in S2 File with a 4 min window. The S1 File also explain how the linearity of calibration, sensitivity LOD, LOQ, working ranges, matrix effect, reproducibility, precision and selectivity of the standards and milk proteins were computed.

Accession number, AA sequences and processing information of the milk protein standards were retrieved from UniprotKB knowledge database (last modified 28 August 2015; http://www.uniprot.org/). AA sequence were then manually modified to account for protein maturation processes including signal peptide cleavage and post-translational modifications (PTMs) such as phosphorylation, glycosylations, and allelic variations using information from both UniprotKB and report from [7]. This investigation is summarised in Table B in S2 File.

All relevant data are within the paper and the stable public repository MassIVE. Data at MassIVE are hosted at the following URL with corresponding Accession Number: URL: http://massive.ucsd.edu/ProteoSAFe/datasets.jsp Accession Number: MSV000080036.

## 3. Results and Discussion

### 3.1. Optimisation of HPLC separation of bovine protein external standards

**Fig 2:**
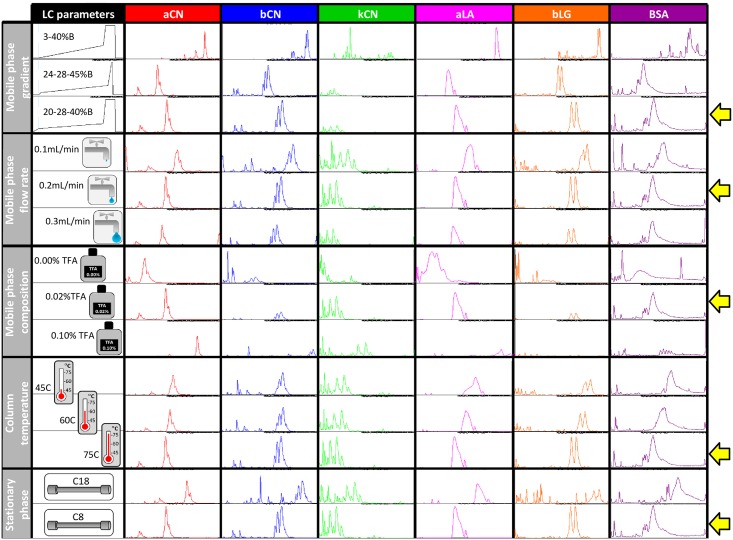
Visual summary of the HPLC optimisation. HPLC separation was optimised by modifying the gradient, flow rate and composition of the mobile phases as well as testing different temperatures and stationary phase chemistries of the separation column. The first column lists the various conditions tested and the following columns display the results for each external standard analysed in this study. Base Peak Chromatograms (BPCs) are displayed from 2.5 min to 32.5 min on the x axis (retention time). The same intensity scale was displayed on the y axis for a given standard and parameter. Yellow arrows on the right hand side point to optimum conditions for each parameter tested. aCN, alpha casein; bCN, beta casein; kCN, kappa casein; aLA, alpha lactalbumin; bLG, beta lactoglobulin; BSA, bovine serum albumin.

Fig 2 summarises our HPLC test results and the yellow arrows point to the conditions that were deemed optimum in our hands.

#### 3.1.1. Impact of gradient, flow rate, and composition of the mobile phase

In our study, three gradients were tested in which not only the starting conditions differed (3, 20 or 24% phase B) but also the ramping steps during which protein elution occurred (3–40%, 28–45%, or 28–40% phase B). When the 3–40% B gradient was employed, most proteins eluted during the second half of the separation run (15–32 min, Fig 2). One exception was kCN which displayed the earliest RT and eluted throughout the run. Also worth noting is the highest base peak from kCN standard was 4.5 more intense under 3–40% B gradient than when the other two gradients were applied. This gradient usually applies to peptide separation by RP-HPLC [26]. Because whole proteins are much longer than peptides therefore more hydrophobic, eluting them from the stationary phase therefore requires higher organic solvent concentrations. As more than half the separation time was not exploited, 3–40% B gradient was deemed unsuitable. The 24-28-45% B gradient was based on method fromBobe *et al*. [13] and applied more concentrated solvent condition both at the start of the run and the end of the separation step than that of 3–40% B gradient. This gradient generated HPLC profiles in our hands comparable to [13], except for aLA which eluted earlier under our conditions. The protein standards mostly eluted during the first half of the separation run (2.5–20 min) and therefore the second half of the run was not efficiently exploited. Consequently we did not select this 24-28-45% B gradient. The 20-28-40% B gradient was a variation of 24-28-45% B gradient in which solvent concentration both at the start of the run and the end of the separation step was slightly lowered to slow protein elution down. Indeed, overall elution with 20-28-40% B gradient occurred from 5 to 25 min and peaks were visually more intense and narrower than those under 24-28-45% B gradient. Based on these results, 20-28-40% B gradient was selected for our HPLC method.

Applying 20-28-40% B gradient, three flow rates were evaluated at 0.1, 0.2, or 0.3 mL/min. As expected, the greater the flow rate, the quicker the elution of protein standards (Fig 2). Furthermore, the quickest flow rate compromised peak intensity, whilst the slowest flow rate negatively affected peak shape and narrowness. Therefore, the intermediate flow rate of 0.2 mL/min was selected for our method. Our rationale was to minimise the volume of solvent used to enhance ionisation and also reduce the cost of the analysis but without compromising the quality of protein separation. Apart from [14] and [20] who applied a 0.25 and 0.20 mL/min flow rate respectively, generally, faster flow rates have been applied from 0.4 mL/min [21], 0.5 mL/min [15], 0.8 mL/min [18], 1.2 mL/min [13] to 3.0 mL/min [22]. For the comparison to be accurate, HPLC column dimensions, and particle and pore sizes must also be taken into account (Table C in S2 File). Column efficiency is often used to compare the performance of different columns. Efficiency ranged from 25 to 14% in the articles cited here, with our C8 sitting in the middle with an efficiency of 20.8%.

Applying 20-28-40% B gradient and 0.2 mL/min flow rates, we tested the addition of TFA to our mobile phases A (H_2_O/0.1% FA) and B (ACN/0.1%FA). Three concentrations were employed 0, 0.02, and 0.1% TFA. Signal intensity was systematically the lowest with 0.1% TFA, symptomatic of in-source ion suppression, for all standards; moreover elution was delayed by several minutes (Fig 2). When 0.02% TFA was added to the mobile phases, the intensity of bCN and bLG was affected, with the intensity of the other standards remaining unchanged. Consistently, peak shape and narrowness were greatly improved with 0.02% TFA compared to no TFA at all, and RTs were not affected. When no TFA was added to the mobile phases, proteins eluted during the first half of the separation run. Based on these observations, it was decided to include 0.02% TFA to our mobile phases for all subsequent LC-MS run. Traditionally intact milk proteins have been detected by chromatography where high concentrations of TFA (0.1%) in both mobiles phases A and B have been used (Table C in S2 File; [14–19, 22]). TFA, a strong pairing agent that mitigates cation exchanges during HPLC separation, improves the chromatographic separation of proteins by increasing the solubility of eluted proteins in ACN [27]. High concentrations of TFA are not recommended when MS analyses are to be performed as this strong acid severely suppresses analyte ionisation in the ESI source. Aware of this phenomenon, TFA concentration in mobiles phases was dropped to 0.01% [21], thus ensuring successful identification of aS1CN and bCN variants by MS.

#### 3.1.2. Effect of column temperature and chemistry

Applying 20-28-40% B gradient, 0.2 mL/min flow rates, and 0.02% TFA to the mobile phases, we then turned our attention to the separation column by first testing three distinct oven temperatures: 45, 60 and 75°C. As expected, the higher the temperature, the quicker the elution of protein standards, particularly when 75°C was applied (Fig 2). Both peak intensities and shapes were superior at 75°C relative to 45 or 60°C. Therefore, 75°C was selected as our optimum temperature. The temperature of the RP-HPLC column plays an important role in the separation of intact proteins as it affects both protein conformation and mass transfer kinetics; high temperatures maintain protein denatured states [28]. The column we used offered a broad range of temperatures, being stable at up to 90°C. Previous publications did not apply such high temperatures (Table C in S2 File); column temperature ranged from ambient [13, 18], 35°C [16, 17], 40°C [14, 22], 45°C [15], to 50°C [21].

For determination of separation efficiency based on column chemistry, two different stationary phases from the same supplier were evaluated; a C18 column usually applied to peptide separation and a C8 column, more commonly used for intact protein separation. Not only were these columns packed with distinct stationary phases, but also had different particle size; thus bearing different column efficiencies (N = 20.8% for the C8 column and N = 44.1% for the C18 column), hence different resolutions. Their dimensions and pore sizes were the same, thus displaying equivalent interstitial or dwell volumes (286 μL). With the temperature set at 75°C, the columns produced vastly different separation profiles (Fig 2). This was expected considering the C18 column displays more than twice the resolving power of the C8 column. Using a C18 column changed the BPCs of the standards to such an extent, in particular for bCN, kCN, and bLG, that we could no longer compare them to published results [13–18, 21,24]. Moreover, deconvoluted masses of these additional peaks obtained using the DISSECT, Maximum Entropy and SNAP algorithms did not correspond to known proteins (data not shown). Identifying such proteins would require top-down sequencing experiments, which is beyond the scope of this paper. Consequently, we selected C8 chemistry. When a C18 chemistry was employed with UV detection to separate milk major intact proteins (Table C in S2 File), aS1CN-9P phosphorylated form and bCN A1 and A2 variants could not be resolved [13, 22]. A C8 chemistry (Zorbax 300SB-C8 RP, 3.5 μm particle, 300 Å pores, 150 × 4.6 mm, Agilent Technologies) with UV detection was successfully employed to resolve all major casein variants [15], in an elution order very similar to that described here, with the exception of aLA which eluted between bCN A2 and bLG B. The same Aeriswidepore XP-C8 chemistry employed here was also used [21] albeit with smaller column dimensions (2 x 100 mm) and identical particle size (3.6 μm), followed by MS analysis; aLA eluted between aS1CN-9P and bCN A1 similarly to our chromatograms.

### 3.2. Optimisation of MS analysis using bovine protein external and internal standards

#### 3.2.1. Impact of the mass scanning rate and window

**Fig 3:**
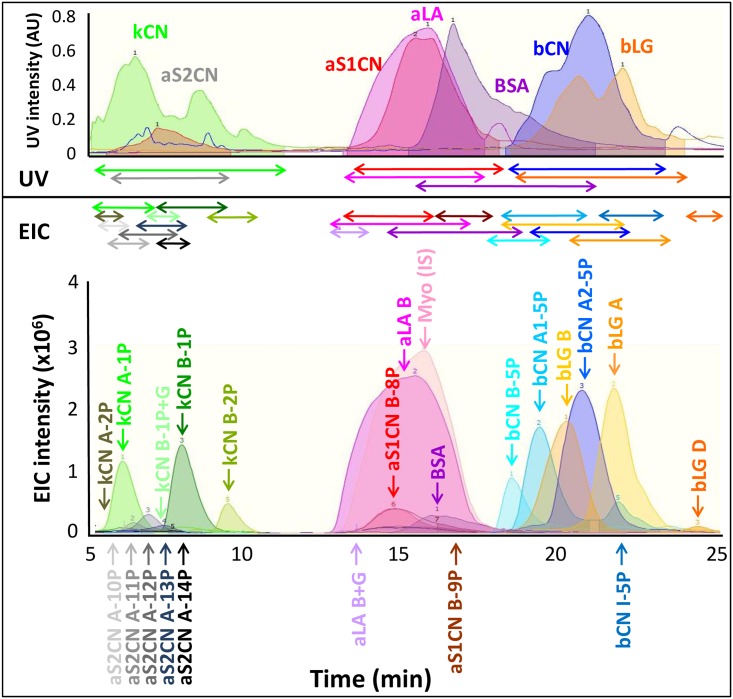
UV traces at 214 nm and EICs over time (5–25 min) of external protein standards. Standards were prepared at the same concentration, run independently and overlaid to illustrate that ionisation efficiency varied from one protein to the other. All external standards purchased from Sigma (aCN, bCN, kCN, aLA, bLG, BSA, and myoglobin) were dissolved in 50% Solution A to a 10 mg/mL concentration. The coloured arrows in between the UV traces and the EICs represent the elution windows of the bovine protein standards.

Two scanning rates were tested, 0.7 Hz (one scan every 1.5 seconds) or 0.3 Hz (one scan every 3.0 seconds). Peak intensities doubled when using the 0.3 Hz scanning rate which was at half the speed as the 0.7 Hz rate (Fig A in S2 File). Another anticipated consequence was that the number of data points recorded along the chromatogram was halved at 0.3 Hz scanning rate relative to 0.7 Hz rate. As some standards do not ionise efficiently (e.g. aCN and BSA, Fig 3), thereby considerably diminishing peak intensity, the setting that favoured intensity over data point density was selected (*i*.*e*. 0.3 Hz) for our method. This method allowed a minimum of 20 data points to be collected across each peak (Fig 3), which was sufficient for quantitation.

Applying a 0.3 Hz scanning rate, mass scanning range of the MS was evaluated by scanning either from 600 to 6000 m/z or from 600 to 3000 m/z. In order to visualise the richness of the spectral signal along the whole mass range, for each standard the BPC was averaged from 5 to 25 min to produce an averaged mass spectrum. Examination of the 3000–6000 m/z range showed very little spectral signal with our MS ion transfer settings (Fig A in S2 File), therefore 600–3000 m/z scan range was selected for our method.

#### 3.2.2. Mass resolution and accuracy, and identification of PTMs

Isotopic patterns were obtained for all proteins of interest (Fig B in S2 File), except BSA whose high MW prevented isotope resolution and for which average mass was therefore retrieved. Deconvolution using the Maximum Entropy algorithm resulted in monoisotopic masses (except for BSA) with resolution ranking from 33522 (bCN I-5P) to 48843 (aS2CN A-13P), FWHM between 0.7145 (bCNI-5P) and 0.4114 (aLA B+G) (Table 1). Based on these results, we can confidently conclude that the Q-TOF instrument employed in this study generated highly resolving mass spectra.

**Table 1:** MS parameters for each milk protein external standards and myoglobin internal standard following mass deconvolution.

Protein code	Observed monoisotopic mass (Daltons)	Resolution	S/N	FWHM	Theoretical monoisotopic mass (Daltons)	Mass difference (Daltons)	Error (ppm)
aLA B	14176.8143	33687	666399	0.4208	14176.798	-0.0163	-1.15
aLA B+G	14500.9142	35248	90016	0.4114	14500.902	-0.0124	-0.86
aS1CN B-8P	23600.2457	39446	119684	0.5983	23600.472	0.2263	9.59
aS1CN B-9P	23680.2289	39607	45101	0.5979	23680.472	0.2431	10.27
aS2CN A-10P	25133.0447	48319	11684	0.5202	25133.343	0.2983	11.87
aS2CN A-11P	25213.0404	42536	26278	0.5927	25213.343	0.3026	12.00
aS2CN A-12P	25292.9669	48114	9865	0.5257	25293.343	0.3761	14.87
aS2CN A-13P	25372.9424	48843	14009	0.5195	25373.343	0.4006	15.79
aS2CN A-14P	25451.8891	47390	4572	0.5371	25453.343	1.4539	57.12
bCN A1-5P	24008.2085	38719	119441	0.6201	24008.317	0.1085	4.52
bCN A2-5P	23968.2044	38068	203125	0.6296	23968.311	0.1066	4.45
bCN B-5P	24077.2559	40473	51676	0.5949	24077.386	0.1301	5.40
bCN I-5P	23950.2291	33522	70663	0.7145	23950.355	0.1259	5.26
bLG A	18354.4897	37757	353954	0.4861	18355.446	0.9563	52.10
bLG B	18269.4593	38485	293317	0.4747	18269.409	-0.0503	-2.75
bLG D	18268.4292	40301	23874	0.4533	18268.410	-0.0192	-1.05
BSA*	66462.5929	22050	2645	3.0142	66462.966	0.3731	5.61
kCN A-1P	19026.5498	36763	166573	0.5175	19026.542	-0.0083	-0.44
kCN A-2P	19106.4925	37941	83630	0.5036	19106.542	0.0495	2.59
kCN B-1P	18994.5907	36940	184866	0.5142	18994.589	-0.0022	-0.12
kCN B-1P+G	19650.8391	42612	18763	0.4612	19650.817	-0.0224	-1.14
kCN B-2P	19075.5445	36427	2982	0.5237	19074.589	-0.9555	-50.09
Myo (IS)	16940.9974	37215	74077	0.4552	16940.956	-0.0414	-2.44

These parameters were exported from DataAnalysis Spectrum Data window where Observed monoisotopic mass is the deconvoluted mass using Maximum Entropy algorithm experimentally recorded in Daltons, Resolution is the mass resolution of the deconvoluted spectra, S/N is the signal-to-noise ratio of the deconvoluted spectra, Intensity is the intensity of the most abundant ion in the deconvoluted spectra, and FWHM is the full width at half maximum. The theoretical monoisotopic masses were computed using the online Peptide Mass Calculator tool from Peptide Protein Research Ltd. (http://www.peptidesynthetics.co.uk/tools/). Mass accuracy was assessed by subtracting the observed deconvoluted masses to the theoretical ones and registered in Daltons in the Mass difference column, and further converted to parts per million error in the Error (ppm) column.

* average mass.

Theoretical masses were obtained using manually curated sequences (Table B in S2 File) and when compared to the observed masses, the mass difference was always less than one Dalton, bar aS2CN A-14P (Table 1). Most protein standards displayed less than 0.4 Da (or 16 ppm) error and as little as -0.44 (kCN A-1P); except kCN B-2P (-0.95 Da or -50 ppm), bLG A (0.95 Da or 52 ppm), and aS2CN A-14P (1.45 Da or 57 ppm). Caution should be taken when claiming the presence of aS2CN A with 14 phosphorylated groups in the external standards and milk samples, as its mass error is greater than 1 Da and this particular phospho-form of aS2CN has not been reported in the literature. Its presence in milk must be validated experimentally.

By cross-checking protein deconvoluted masses with public data sources (uniprotKB; [7–10]) we were able to reliably identify the milk protein variants and some of their phosphorylated and glycosylated proteoforms (Table 1). The use of external protein standards, allowed the detection of variant B of aS1CN with 8 and 9 phosphorylations, variant A of aS2CN with 10 to 14 phosphorylations, variants A1, A2, B and I of bCN with 5 phosphorylations, variant A of kCN with 1 and 2 phosphorylations, variant B of kCN with 1 and 2 phosphorylations and a glycosylation of 656 Da (GalNAc-Gal(NeuAC)), variants A, B and D of bLG, variant B of aLA with or without a glycosylation of 324 Da, as well as the BSA variant that bears a threonine residue at position 224 of the AA sequence instead of an alanine residue. Apart from BSA at 66.5 kDa, major proteins from bovine milk are of medium MW, ranging from 14.2 kDa (aLA B) to 25.4 kDa (aS2CN A-14P). An ESI-Q-TOF MS platform was also employed in [18, 20]. A simple TOF instrument was used to identify cow’s milk proteins based on deconvoluted mass information [21]. Using the Microtof QII high resolution mass spectrometer with a 20,000 resolving power and 2 ppm mass accuracy (Bruker Daltonics Gmbh), monoisotopic masses of milk caseins were obtained and variants A1, A2, B and C of bCN using specific ions, such as 22+ charge state, to produce EICs were thus quantified [20].

#### 3.2.3. Determination of protein ionisation efficiency

UV traces of external standards made up at the same concentration (e.g. 10 mg/mL) and obtained during RP-HPLC separation were comparable across standards, with bLG and aS2CN displaying lower intensities (Fig 3, top panel). However, EICs of external and internal standards made up at the same concentration did not produce similar intensity and peak shape patterns (Fig 3, bottom panel). This demonstrates that ionisation and transmission efficiencies vary from one protein to the other. Ionization efficiency is the effectiveness of producing gas-phase ions from analyte molecules in solution within the ESI source and transmission efficiency is the ability to transfer the charged species from atmospheric pressure of the ESI source to the low-pressure region of the mass analyzer [29]. The efficiency at which ions are being ionised varies with their mobility, which differs among ion species [30]. Based on obtained peak areas from their corresponding EICs, the proteins ranked as follows: Myo>aLA B >bCN A2-5P = bLG A >bLG B = bCN A1-5P >kCN B-1P >kCN A-1P >bCN B-5P >kCN B-2P >bCN I-5P > aS2CN A-12P > aS1CN B-8P > BSA > aS1CN B-9P >bLG D > aS2CN A-11P > aS2CN A-13P > aS2CN A-10P > aS2CN A-14P >kCN B-1P+G = kCN A-2P = aLA B+G. The bovine protein most abundant in milk, aS1CN, displayed the least efficient ionisation under our ESI conditions. This illustrates one artefact of MS as ion chromatograms do not necessarily reflect the abundance of a given protein in a sample but rather how ionisable this component is. This is why calibration curves of external standards at increasing concentrations are essential to quantify known proteins using MS. The horizontal arrows in Fig 3 visually illustrate that due to extensive overlap of milk protein variants, UV trace alone is not suitable to reliably integrate their individual peak areas for quantitative purpose. By further discriminating intact proteins according to their m/z, MS offers an additional orthogonal separation level to HPLC, both of which complementing each other to individualise major bovine protein variants.

### 3.3. Method validation

#### 3.3.1. Calibration, matrix effect and sensitivity

Using our optimum MS scanning rates and mass window, calibration curves were produced in duplicate along a 0.1–10.0 mg/mL concentration range for each external standard. Fig C in S2 File further exemplifies how ionisation efficiency varied from one standard to the other. Overall, linear curves were obtained and positively highly correlated with increasing concentrations of analytes (R^2^ ranking from 0.97 for BSA to 0.99 for aCN). LODs ranked from 0.46 mg/mL (aCN) to 2.10 mg/mL (BSA) and LOQs ranked from 1.50 mg/mL (aCN) to 7.01 mg/mL (BSA) (Table 2). Based on these results, the working ranges (0.9 to 10 mg/mL) covered most of the concentration range tested in our study in bovine protein standards.

**Table 2:** Response using EIC peak areas of each external standards over increasing concentrations.

	aCN (S1+S2)		bCN (A1+A2+B+I)		kCN (A+B)		aLA (B, B+G)		bLG (A+B+D)		BSA	
Concentration	Mean	SD	Mean	SD	Mean	SD	Mean	SD	Mean	SD	Mean	SD
0.25	0.261	0.020	0.812	0.099	0.590	0.016	2.823	0.082	0.719	0.010	0.371	0.031
0.50	0.651	0.015	1.808	0.201	1.259	0.023	4.248	0.131	1.759	0.041	0.683	0.082
0.75	1.037	0.020	3.523	0.324	1.977	0.014	6.598	0.157	2.832	0.058	1.061	0.102
1.00	1.484	0.035	5.103	0.444	2.920	0.007	10.825	0.236	3.910	0.116	1.213	0.204
2.50	2.893	0.086	13.423	0.603	6.693	0.101	21.581	0.340	8.306	0.246	2.268	0.306
5.00	5.483	0.191	21.769	1.121	11.697	0.317	36.286	0.540	17.363	0.479	3.309	0.408
7.50	8.028	0.311	28.721	1.521	15.247	0.585	50.038	0.626	23.026	0.693	4.337	0.510
10.00	11.053	0.396	33.320	1.896	18.672	0.891	58.970	0.789	27.211	0.913	5.001	0.612
***SLOPE***	*1*.*074*		*3*.*447*		*1*.*882*		*5*.*916*		*2*.*814*		*0*.*469*	
***SE***	*0*.*163*		*2*.*360*		*0*.*943*		*2*.*963*		*1*.*376*		*0*.*329*	
***INTERCEPT***	*0*.*170*		*1*.*709*		*0*.*913*		*3*.*583*		*0*.*969*		*0*.*668*	
***R***^***2***^	*0*.*999*		*0*.*971*		*0*.*984*		*0*.*984*		*0*.*985*		*0*.*970*	
***LOD (mg/mL)***	*0*.*457*		*2*.*054*		*1*.*504*		*1*.*502*		*1*.*468*		*2*.*102*	
***LOQ (mg/mL)***	*1*.*522*		*6*.*846*		*5*.*012*		*5*.*008*		*4*.*892*		*7*.*006*	
***Working range***	*0*.*5–10 mg/mL*		*2*.*0–10 mg/mL*		*1*.*5–10 mg/mL*		*1*.*5–10 mg/mL*		*1*.*5–10 mg/mL*		*2*.*1–10 mg/mL*	

External standards were run in duplicates. Based on the averaged results, the slope, standard error (SE), intercept, Pearson correlation coefficient (R^2^) values, limits of detection (LOD) and quantitation (LOQ), and working range were computed. LOD for each standard was obtained using the following formula: 3*(standard error/slope). LOQ for each standard was obtained using the following formula: 10*(standard error/slope). The working range was the interval between the LOQ and the upper concentration of the analyte in the samples tested in this study (10 mg/mL) for which linearity was demonstrated.

The effect of the matrix was tested by spiking the internal standard protein myoglobin at increasing concentrations (0.1–10 mg/mL) into three different matrices: 1/ 50% Solution A which is used to prepare the milk samples for LC-MS analysis and was our control, 2/ a protein sample prepared from Jersey skim milk, and 3/ a protein sample prepared from Holstein skim milk. Trend lines on Fig D in S2 File demonstrated the linearity of myoglobin response along the concentration range, irrespective of the matrix used, with high reproducibility. High reproducibility was further confirmed numerically in Table 3 with a coefficient of variation (CV) well below 10% for both RTs and responses, irrespective of the matrix used.

**Table 3:** Averaged RTs and response of myoglobin internal standard prepared in Solution A or spiked in milk matrices a cross 2 technical replicates.

		Retention time			Response (Peak area)				
matrix	concentration (mg/mL)	Average (min)	SD (min)	CV (%)	Average	SD	CV (%)	matrix effect (%)	S/N
solution A	0.00	18.14	0.26	1.42	8336	548	6.57	0	1
solution A	0.10	17.69	0.04	0.20	10841067	273937	2.53	0	1158
solution A	0.20	17.69	0.04	0.20	18803577	1063155	5.65	0	2043
solution A	0.25	17.55	0.03	0.16	25659384	1028574	4.01	0	2729
solution A	0.50	17.44	0.06	0.37	49743658	1288018	2.59	0	5343
solution A	0.75	17.28	0.04	0.20	69296004	1950636	2.81	0	7453
solution A	1.00	17.25	0.00	0.00	89528068	3681300	4.11	0	9729
solution A	2.50	16.91	0.10	0.59	187397208	8633480	4.61	0	19980
solution A	5.00	16.57	0.06	0.38	309549728	2783424	0.90	0	33225
solution A	7.50	16.41	0.03	0.17	415503536	4933296	1.19	0	44772
solution A	10.00	16.07	0.00	0.00	617516960	7595616	1.23	0	66266
Jersey sample	0.10	17.39	0.25	1.46	9740110	56148	0.58	-10.16	983
Jersey sample	0.20	17.46	0.03	0.16	17026691	128301	0.75	-9.45	1729
Jersey sample	0.25	17.43	0.04	0.20	21101578	162358	0.77	-17.76	2168
Jersey sample	0.50	17.18	0.06	0.33	40613736	272784	0.67	-18.35	4173
Jersey sample	0.75	17.11	0.04	0.21	57280948	65996	0.12	-17.34	5930
Jersey sample	1.00	17.02	0.06	0.33	85657548	3627620	4.24	-4.32	8686
Jersey sample	2.50	16.75	0.25	1.52	168809992	3279336	1.94	-9.92	17764
Jersey sample	5.00	16.64	0.10	0.59	282883424	4637792	1.64	-8.61	30279
Jersey sample	7.50	16.43	0.10	0.61	381639168	5000000	1.31	-8.15	41390
Hosltein sample	0.10	17.55	0.10	0.56	9118925	810674	8.89	-0.28	934
Hosltein sample	0.20	17.46	0.03	0.16	18097231	1448261	8.00	-7.25	1899
Hosltein sample	0.25	17.39	0.07	0.41	24715424	1993082	8.06	-5.54	2521
Hosltein sample	0.50	17.31	0.01	0.08	46471016	2130032	4.58	-15.51	4874
Hosltein sample	0.75	17.16	0.00	0.00	73999076	3770028	5.09	11.58	7827
Hosltein sample	1.00	17.14	0.03	0.17	92522796	7680108	8.30	5.23	9570
Hosltein sample	2.50	16.91	0.03	0.17	188049680	10025248	5.33	0.76	19820
Hosltein sample	5.00	16.69	0.04	0.21	327507568	11226448	3.43	10.64	34420
Hosltein sample	7.50	16.57	0.13	0.77	416283136	21503264	5.17	0.28	44200

Matrix effect was computed by subtracting IS response in milk sample (either Jersey or Holstein samples) to that in Solution A and dividing the difference by the response in Solution A. Results were then converted to percent. Sensitivity was assessed using the signal-to-noise ratio (S/N).

Matrix effect was more pronounced for Jersey samples that for Holstein sample particularly at low concentrations (Table 3), averaging 11.5% and 6.3% respectively. Globally, matrix suppressed Myoglobin ion intensity.

Sensitivity was assessed using the obtained signal-to-noise ratio (S/N). Using triplicate blanks to assess the noise, our results showed a very high S/N, with a minimum of 934, well above the standard LOQ threshold of 10 (Table 3).

Based on Tables 3 and 4 reports the slope, SE, intercept, R^2^, LOD, LOQ and working range of myoglobin calibration curve within each matrix either over the entire concentration range (0–10 mg/mL) or over a range limited to 0–1 mg/mL. Statistics were improved at a lower concentration (Table 4 and inset in Fig D in S2 File) because the linear trend was then a better fit. We chose to spike myoglobin at a 0.2 mg/mL concentration into milk samples because of its low CV, linearity, and LOQ (Table 4). At this concentration, ion suppression was 7% in Holstein sample and 15% in Jersey samples (Table 3).

**Table 4:** Slope, standard error (SE), intercept, Pearson correlation coefficient (R2) values, limits of detection (LOD) and quantitation (LOQ), and working range of myoglobin calibration curve.

RANGE	Myoglobin response (0–10 mg/mL)			Myoglobin response (0–75 mg/mL)		
MATRIX	50% Solution A	Jersey sample	Holstein sample	50% Solution A	Jersey sample	Holstein sample
***SLOPE***	58336520	50745893	56156165	92981539	73818123	98846238
***SE***	19010009	15462981	20733548	1650282	786991	1416302
***INTERCEPT***	15644753	17941811	21909458	1164209	2578088	-1104311
***R***^***2***^	0.9920	0.9882	0.9827	0.9967	0.9988	0.9978
***LOD (mg/mL)***	0.98	0.91	1.11	0.05	0.03	0.04
***LOQ (mg/mL)***	3.26	3.05	3.69	0.18	0.11	0.14
***Working range***	0.9–10 mg/mL	0.8–10 mg/mL	1.1–10 mg/mL	0.05–1 mg/mL	0.03–1 mg/mL	0.04–1 mg/mL

#### 3.3.2. Reproducibility, selectivity, and precision

External standards were run in triplicate, with and without spiked Myoglobin. The EICs of the proteins of interest overlaid very well across all six replicates, irrespective of the presence of IS or not (Fig E in S2 File), thus demonstrating good reproducibility. By using the ion series indicated in Table A in S2 File to produce EIC for each protein of interest, and limiting this EIC to the RT at which the standard is expected for peak integration (shaded area in Fig F in S2 File), we can selectively detect and quantify milk protein standards.

Excellent reproducibility levels are numerically confirmed in Table 5 with all CVs being below 6%. The response CV of external standards solubilised in 50% Solution A was overall smaller when the IS was not spiked into the external standards. Indeed, in the presence of IS, CV varied from 0.1 to 2.7% with an average of 2.3% (+/- 1.4%), whereas in the absence of IS, CV varied from 0.3 to 5.3%, with an average of 1.3% (+/- 0.7%). As expected, normalising the protein response using an IS helped make the data more reproducible.

**Table 5:** Quantitative reproducibility of standards with or without IS across triplicates.

	unormalised (without IS)			normalised with IS		
Protein	Average RT (min)	Average Response	CV Response (%)	Average RT (min)	Average Response	CV Response (%)
aLA-B	15.78	501203008	2.2	15.91	24.1758	2.4
aLA-B-G	13.95	2709020	2.2	14.14	0.1326	2.3
aS1CN-B-8P	15.28	46743521	1.9	15.38	2.2394	3.4
aS1CN-B-9P	17.88	2757610	1.4	17.89	0.1435	3.4
aS2CN-A-10P	6.79	6453168	0.1	6.91	0.2907	4.0
aS2CN-A-11P	7.08	11045553	0.4	7.18	0.5287	5.1
aS2CN-A-12P	7.56	20480763	1.7	7.69	0.9351	2.9
aS2CN-A-13P	8.03	11144854	1.2	8.14	0.5129	4.0
aS2CN-A-14P	8.30	4472310	0.5	8.43	0.2273	5.3
bCN-A1-5P	19.67	144641829	1.6	19.68	6.6732	2.1
bCN-A2-5P	21.12	261552277	2.7	21.20	11.8123	2.1
bCN-B-5P	18.76	58604908	1.4	18.82	2.7343	0.7
bCN-I-5P	22.61	7497135	1.7	22.55	0.2589	2.0
bLG-A	22.09	242632197	1.8	22.19	9.6131	1.0
bLG-B	20.64	195728821	2.3	20.64	7.7325	0.3
bLG-D	24.74	7633926	0.6	24.74	0.2813	0.8
BSA	16.53	56264133	1.5	17.05	4.9275	3.6
kCN-A-1P	6.44	61070224	1.1	6.44	2.7045	0.9
kCN-A-2P	8.39	8329892	0.4	8.43	0.3715	1.1
kCN-B-1P	8.28	78989451	0.8	8.42	3.5399	0.5
kCN-B-1P-G	7.89	4958922	0.9	7.95	0.2106	2.0
kCN-B-2P	9.81	20580644	1.3	9.90	0.9046	1.6
Myo	17.91	19602569	0.8	n.a.	n.a.	n.a.

External standards were prepared at a 10 mg/mL concentration in 50% Solution A. Precision was evaluated across repeated measurement results and expressed by coefficient of variation (CV) of replicate results. Minimum, maximum, average and standard deviation (SD) values across CVs are presented to emphasise the gain in reproducibility when an internal standard (IS) is used. na, not applicable; nd, not detected.

### 3.3. Application to cow’s milk samples

**Fig 4:**
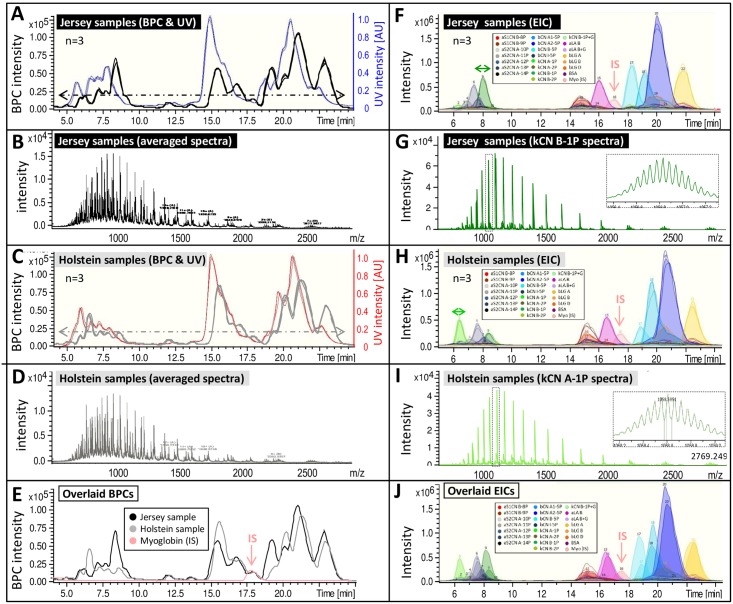
Method validation using milk samples. Optimum method was tested on milk samples (3 replicates) with or without internal standard (IS, myoglobin). Panel A, base peak chromatograms (BPCs) and UV trace at 214 nm of the Jersey bulk milk sample spiked with IS and run in triplicates. Panel B, spectra averaged across 5–25 min (see arrow in panel A) of the Jersey BPC and displayed along the whole m/z (600–3000) range. Panel C, BPCs and UV trace at 214 nm of the Holstein bulk milk sample spiked with IS and run in triplicates. Panel D, spectra averaged across 5–25 min (see arrow in panel C) of the Holstein BPC and displayed along the whole m/z (600–3000) range. Panel E, BPCs of Jersey sample, Holstein sample, and IS overlaid. Panel F, extracted ion chromatograms (EICs) of the Jersey sample spiked with IS and run in triplicates. Panel G, averaged spectra of kCN B-1P (see arrow in panel F) along 600–3000 m/z and zoomed in on the most abundant ion (1056.6 m/z) in inset. Panel H, EICs of the Holstein sample spiked with IS and run in triplicates. Panel I, averaged spectra of kCN A-1P (see arrow in panel H) along 600–3000 m/z and zoomed in on the most abundant ion (1058.6 m/z) in inset. Panel J, overlaid EICs of one Jersey sample replicate and one Holstein sample replicate.

The final validation step for our method was to apply theLC and MS parameters that were deemed optimum for the analysis of external standards on milk samples from two distinct cow breeds, Jersey and Holstein-Friesian (shorten to Holstein in Tables and figures for ease of reading). Milk samples spiked with myoglobin IS were run in triplicates. Milk proteins eluted from 5 to 25 min, with the RT from 10.0 to 14.5 min being protein-poor (Fig 4). All the proteins of interest identified using external standards were successfully detected in the milk samples from Jersey and Holstein-Friesian cows as evidenced by the EICs. UV traces, BPCs and EICs of these proteins were very similar across technical triplicates and therefore overlaid nicely. Fig 4 further illustrates that UV traces and BPCs alone are not sufficiently resolved to allow the quantitation of individual milk protein variants. Variants were individualised by extracting the chromatograms of their corresponding ions, and their abundances (*i*.*e*. response) in milk samples were inferred by integrating the peak areas of the EICs (Table 6). This strategy is schematised in Fig G in S2 File.

**Table 6:** Quantitation of protein variants from milk samples.

	Jersey milk			Holstein milk			T-test	
Protein	Average RT (min)	Average Response	CV Response (%)	Average RT (min)	Average Response	CV Response (%)	p-value Response	significance
aLA-B	16.83	3.1430	2.1924	17.00	2.6865	3.7495	0.0006	***
aLA-B-G	15.61	0.2639	13.5836	15.42	0.1970	6.2683	0.0236	*
aS1CN-B-8P	15.46	1.5917	3.9823	15.61	1.5511	7.5522	0.6165	n.s.
aS1CN-B-9P	16.54	0.4291	6.2196	16.68	0.3363	3.3860	0.0015	**
aS2CN-A-10P	7.12	0.5305	3.8224	6.92	0.5573	6.3023	0.2962	n.s.
aS2CN-A-11P	7.32	0.9330	6.4905	7.43	0.6549	4.4493	0.0004	***
aS2CN-A-12P	7.82	2.2673	1.9770	7.95	1.6973	4.0134	0.0000	***
aS2CN-A-13P	8.13	1.1416	2.4312	8.43	0.8545	5.0902	0.0001	***
aS2CN-A-14P	8.31	0.5240	2.1923	8.66	0.3464	6.9380	0.0000	***
bCN-A1-5P	20.12	5.1872	1.8887	20.16	6.9617	4.9996	0.0001	***
bCN-A2-5P	21.04	12.8962	2.8863	21.19	11.2693	5.2077	0.0067	**
bCN-B-5P	19.22	4.7576	4.2614	19.36	2.0470	1.7351	0.0000	***
bCN-I-5P	23.50	0.0932	2.4644	23.42	0.0987	4.2572	0.2000	n.s.
bLG-A	22.87	5.3522	5.7464	22.98	4.2130	4.3259	0.0015	**
bLG-B	20.85	2.4886	3.4503	20.99	2.8132	4.8285	0.0128	*
bLG-D	23.31	0.1020	5.9428	23.62	0.0847	10.1951	0.0353	*
BSA	15.44	0.9757	4.4888	15.62	0.9283	6.7009	0.3224	n.s.
kCN-A-1P	6.72	0.3971	3.3511	6.67	1.5740	5.4925	0.0000	***
kCN-A-2P	6.41	0.0999	8.1087	6.65	0.1424	4.8685	0.0005	***
kCN-B-1P	8.50	2.7025	3.0301	8.73	0.9285	3.3471	0.0000	***
kCN-B-1P-G	7.30	0.0917	7.0158	7.48	0.1254	12.8383	0.7929	n.s.
kCN-B-2P	9.12	0.1667	8.2451	8.96	0.0920	12.5636	0.0060	**

Milk samples were spiked with myoglobin IS and run using our optimum LC-MS parameters in triplicates. Average retention times (RTs) and normalised responses based on peak area are reported. Precision was evaluated across repeated measurement results and expressed by coefficient of variation (CV) of replicate results. A Student t-test was performed to compare the normalised response of proteins of interest from Jersey milk with that of Holstein milk proteins. n.s. not significant,

* p-value < 0.1,

** p-value <0.01,

*** p-value < 0.001.

When the EICs ofonereplicate from each breed was overlaid one on top of the other, all protein peaks were found and their intensities varied in a breed-specific manner. For instance, kCN B-1P, bCNB-5P, and bCNA2-5P levels were higher in the Jersey sample than in the Holstein sample. Conversely, the levels ofkCN A-1P and bCN A1-5P were increased in the Holstein milk than in the Jersey milk. Averaging mass spectra over the protein elution profile produced ion distributions mostly condensed around 900–1800 m/z, irrespective of the breed (Fig 4). The proteins that varied the most across breeds, namely kCNs, were chosen to exemplify spectra quality (Fig 4). A well-defined charge envelope is visible for the proteins displaying isotopic resolution. Spectra were sufficiently resolved to achieve isotopic separation and therefore deconvoluted into accurate monoisotopic masses for all proteins with the exception of BSA for which average mass was obtained. Reproducibility levels were acceptable as assessed by the CV which ranged from1.7 to13.6% (Table 6). A Student t-test was performed to determine which protein variants differed between the breeds. If we arbitrarily consider a cut-off *p*-value of 0.01, the expression levels of all variants were significantly different, except for aS2CN B-8P, aS2CN A-10P, bCN I-5P, bLG B and D, BSA, and kCN B-1P+G (Table 6). If we drop this cut-off to 0.001, then only aLA B, aS2CN A-11P, 12P, 13P and 14P, bCNA1-5P and B-5P, kCN A-1P and 2P, and kCN B-1P remained significantly affected, with all of them being more abundant in Holstein milk except bCN A1-5P, and all kCN safore-mentioned, whose levels were higher in Jersey milk. Based on these results, we can conclude that our optimum method can be successfully applied to quantify major known milk proteins from two distinct cow breeds.

In bovine milk, the phosphorylation of caseins (which have been extensively characterised) plays in important structural role in the stabilization of calcium phosphate nanoclusters in casein micelles. The total casein fraction in cow milk comprises up to 40% of aS1CN with commonly 8 or 9 phosphorylated serine residues; aS1CN-9P contains an additional phosphorylated serine residue at position 56 of the preprotein [11]. In our study, both phospho-proteoforms were identified; aS1CN B-8P (23600.2457 Da) was distinguished from aS1CN B-9P (23680.2289 Da) with a 79.9832 Da difference. They slightly co-eluted, their apex being less than two minutes apart. In bulk milk from Holstein-Friesian herd, aS1CN B-8P was 2.24 times more abundant than aS1CN B-9P; the trend was reversed in bulk milk from Jersey herd, as aS1CN B-9P was 2.26 times more abundant than aS1CN B-8P. Previous studies using capillary zone electrophoresis reported similar findings; aS1CN-8P form occurred in a 3-fold excess over the aS1CN-9P form in Hostein-Friesian breed [31]. A genome-wide association study revealed that aS1CN-8P and aS1CN-9P were not regulated by the same set of genes, and that lower concentrations of aS1CN-8P were genetically associated with the AA genotype of bLG [11]. Indeed, in our study, Jersey bulk milk which displayed a lesser aS1CN B-8P concentration relative to Holstein-Friesian bulk milk, also displayed a greater bLG A concentration.

Kappa-caseins provide a hydrophilic coating of casein micelles thus preventing micelle association and aggregation, and stabilizing their structure. Out of the 13 kCN variants reported so far, A and B variants dominate Jersey and Holstein-Friesian herds in Denmark [32]. Cows with predominant kCN variant B have been consistently associated with desirable coagulation properties in the cheese-making process. Two phosphorylation sites are known in kCNs; serine residues at positions 148 and 170 of the preprotein sequence; the latter being constitutively phosphorylated while the former is only partially phosphorylated [33]. In our study, both phospho-proteoforms were identified; kCN B-1P (18994.5907 Da) was distinguished from kCN B-2P (19075.5445 Da) with a 80.9538 Da difference. This variation in degree of phosphorylation also altered the elution of the protein with kCN B-1P eluting before kCN B-2P. A glycosylated form of kCN B-1P was also identified (19650.8391 Da) which, based on literature, would bear a GalNAc-Gal-(NeuAC) O-linked oligosaccharide group (656.2484 Da). Variant B of kCN would be more extensively glycosylated than variant A [12], as we observed. This kCN B-P+G variant was also identified using 2-DE and MALDI-TOF MS [33]; where phosphorylation at Ser170 and its glycosylation at Thr152 were characterised [34]. The other glycoprotein identified in our study was aLA B+G variant (14500.9142 Da) with a gain in mass of 324.1 Da, which would correspond to two mannose, and/or glucose and/or galactose residues. To our knowledge, this is the first time this glyco-form of aLA is reported. In fresh cow’s milk, a small fraction of aLA molecules are N-glycosylated at Asn71 which is a rare protein N-glycosylation site [35]. Monosaccharide analyses of glyco-forms of aLA have revealed varying amounts of N-acetylglucosamine(GlcNAc), N-acetylgalactosamine (GalNAc), mannose (Man), galactose (Gal), fucose (Fuc), and N-acetylneuraminic acid (NeuAc), the quantities of Gal, Fuc, and NeuAc being relatively low [36]. This would suggest that the glyco-form of aLA identified in our study would possibly present two mannose residues; such findings evidently need to be confirmed by further experiments. The presence of 14 glycosylated forms of aLA, spanning from 15841.1 to 16685.3 Da have been evidenced in bovine milk [36].

## Conclusions

In this study, we tested several RP-HPLC and MS parameters to optimise the analysis of intact proteins from bovine milk. The optimum quantitative method was successfully applied to two bulk milk samples from different breeds, Holstein-Friesian and Jersey to assess differences in protein concentration. For instance, kCN B-1P was significantly higher in Jersey milk relative to Holstein-Friesian milk; the trend was the opposite for kCN A-1-P. We are currently applying this method in a high-throughput fashion to numerous samples of milk from both breeds to study the impact of lactation cycle, diet regimes and genetic background. The method could also be incorporated into breeding programs to select cows displaying desirable protein variants and content for specific products e.g. cheese manufacturing or evaporated powdered milk purposes. Finally this method is not restricted to raw bovine milk since we have successfully applied it to UHT cow’s milk, full cream goat milk and raw human milk samples (data not shown).

## Supporting Information

S1 File Further background information pertaining to milk proteins, mass spectrometry and PTMs, along with the detailed methods employed in this study and the observation that the elution order of the protein variants does not follow the GRAVY index. (PDF)

S2 File Supporting Figures and Tables. (PDF)

## Abbreviations

aCN: alpha casein
ACN: acetonitrile
aLA: alpha lactalbumin
AA: amino acids
BPC: base peak chromatogram
bCN: beta casein
bLG: beta lactoglobulin
BSA: bovine serum albumin
CV: Coefficient of Variation
EIC: extracted ion chromatogram
ESI: electrospray ionisation
FA: formic acid
kCN: kappa casein
IS: internal standard
LOB: Limit of Blank
LOD: Limit of Detection
LOQ: Limit of Quantitation
MS: mass spectrometry
MW: molecular mass
m/z: mass-to-charge ratio
PA: peak area
PTMs: post-translational modifications
RP-HPLC: reversed-phase high performance liquid chromatography
RT: retention time
R^2^: Pearson correlation coefficient
SD: standard deviation
SE: standard error
TFA: trifluoroacetic acid
UV: ultra violet
