# Supplementary material for: Quantitation and Identification of Intact Major Milk Proteins for High-Throughput LC-ESI-Q-TOF MS Analyses
Source: PLoS One. 2016 Oct 17;11(10):e0163471. doi: 10.1371/journal.pone.0163471 (PMC5066972; doi:10.1371/journal.pone.0163471)
Supplement: S1 File — (PDF) [file pone.0163471.s001.pdf]

Supplementary information for the article entitled
"**Quantitation and identification of intact major milk proteins for high-throughput LC-ESI-Q-TOF MS analyses**."
by Vincent et al. 2016

1. Background information

1.1. Milk proteins

Most of bovine milk, including caseins and whey proteins, are synthesized within the secretory epithelium of the mammary gland and secreted into the milk pool within the alveolar lumen [1]. Owing to their nutritional importance and physicochemical properties, proteins are the main functional components in milk. By altering processing steps during manufacture, the dairy industry generate a vast range of milk protein products, which find various applications in food systems; for instance, solubility in beverages, emulsification in coffee whitener and salad dressings, foaming in whipped toppings, shakes, and mousses, water binding in bread, meats, and sauces, heat stability in UHT milk and custard, gelation in curds, cheese, and yogurt; and acid stability in acid beverages, and fermented drinks [2]. The study of milk protein variants can be applied to breed characterization, diversity, and phylogeny. Furthermore, because milk proteins are involved in various aspects of human diet, characterising the occurrence of alleles associated with a reduced content of different caseins might be exploited for the production of hypoallergenic milk [3].

In Australia, milk production is concentrated in the temperate zones of the south-east states (New South Wales, Victoria, South Australia) where it remains strongly seasonal, reflecting the predominant pasture-based feeding practices. Australian dairy represents a $13 billion farm, manufacturing and export industry, with over 6,000 dairy farmers producing around 9.7

billion litres of milk a year (www.dairyaustralia.com.au). Over the past 10 years, Victoria state-wide has contributed to the national milk production at an average of 65%, and the Gippsland region on its own accounted for 21% of the national production. Protein content is steadily increasing having gained 0.08% in Australia over the last 10 years (going from 3.29 to 3.38%). Milk from Victoria contains on average 0.02% more protein than Australia-wide (www.dairyaustralia.com.au). Imported in Australia in the mid 1800's the Holstein-Friesian was the first dairy breed and now makes up more than 69% of commercial milking cows, while the Jersey breed, introduced in 1829, accounts for 14% of the Australian dairy population.

1.2. Mass spectrometry

Various mass spectrometersare commercially available and vary in their range of applications; for intact protein analysis, the most important factors to consider are mass resolution and accuracy. Resolution is defined as a unit-less ratio of mass divided by the peak width and is typically measured at full-width half maximum (FWHM). Following ESI, protein ions produce complex multi-charged mass spectra displaying a heterogeneity of charge states.These spectra are characterized by an almost normal distribution of intensities around the base peak and increased spacing between the peaks with increasing mass-to-charge ratio (m/z) value. As the m/z value increases, each peak represents one less charge than its immediately adjacent neighbour of lower m/z. Each peak represents an intact protein molecule with some number of adducted protons. High mass resolution is when the isotope peaks are separated. In this instance, it is possible to deduce the monoisotopic ^{12}C peak, and therefore calculate the accurate mass of compound using monoisotopic elemental masses. This is performed by employing a deconvolution algorithm which mathematically transforms these highly-resolved multi-charged peaks into a singly charged peak located on an m/z scale at the molecular mass of the parent compound [4]. If spectra are of low resolution, then a

centroid of a broad unresolved peak is going to be closest to the protein mass calculated using average elemental mass, which is only the best estimate of the molecular mass of the protein [4].

In this study, we used a quadrupole time-of flight (Q-TOF) mass spectrometer (MS) with an ESI source to detect and quantify intact proteins from cow milk. Q-TOF instruments offer a broad mass scanning range, meaning they can measure ions with high m/z, a very beneficial feature for the analysis of large molecules such as intact proteins. Furthermore, Q-TOF MS maintain the integrity of ions as they fly into the mass analyser, another valuable feature for studying intact proteins whose identities are derived from the accurate mass of the unbroken molecule. Q-TOF instruments determine m/z extremely quickly because the ions reach the detectors in microseconds, meaning that the scanning rate can be very fast if needed, albeit at the expense of sensitivity. This is very relevant when the MS is online with the HPLC and chromatographic peaks are narrow; sufficient data points are obtained across the peak thereby ensuring reliable quantitation. The scanning rate can be fine-tuned in order to record enough data points without compromising sensitivity. However, the resolving power of Q-TOF instruments is relatively medium compared to the high resolution offered by Orbitrap instruments or Fourier transform ion cyclotron resonance instruments; Q-TOF MS are therefore less adept at resolving ions of similar m/z. This can impact the study of proteins of large molecular weight such as BSA (~66.5 kDa) as their isotopes cannot be resolved and average mass, instead of isotopic mass, must be used for identification purposes. However, Q-TOF platforms are able to routinely deduce highly accurate average masses, as we demonstrated in this study with the identification of BSA with a 0.37 Da (5.6 ppm) average mass accuracy. Recently, using a Bruker MaXis 4G high resolution Q-TOF at 80,000 resolution, mass deviations well below 1 ppm while analysing intact monoclonal antibodies were reported [5].

1.3. Post-translational modifications of milk proteins

Post-translational modifications (PTMs) can be mathematically assigned by observing mass shifts. For instance, phosphorylation can readily be speculated when a mass shift of +80 Da is observed, which corresponds to the addition of one phosphate group. However, only accurate mass determination is able to conclusively deduce this as being a phosphorylation, in order to discount near isobaric PTMs such as sulfation. Comprehensive characterisation of known protein constituents in milk based on published reports is exploited to support the assignment of phosphorylation. Our literature survey and search through Uniprot database (http://www.uniprot.org/) failed to retrieve publications reporting sulphated forms of caseins, therefore a +80 Da mass shift can reasonably be attributed to the presence of a phosphorylated group. Glycosylation modifications are more diverse and thus can be more difficult to infer from deconvoluted information, depending on the degree of heterogeneity. However, by exploiting known information regarding glycan synthesis in mammalian cells, expected compositions can be deduced. As such, mass shifts can be attributed to specific glyco- forms of proteins. Mass spectrometric methods applied to milk have been reviewed [6]. Following RP-HPLC fractionation of intact proteins from a supermarket bovine milk sample, a dual ESI source coupled to a Time-of-Flight (TOF) MS, which only offers medium resolution hence average masses, was employed to identify aS1CN-8P (23614 Da) and its phosphorylated variant aS1CN-9P (23694 Da) thus detecting a 80 Da average mass difference, as well as variants A1 (23982 Da) and A2 (24022 Da) of bCNs thus detecting a 40 Da average mass difference[7]. A medium resolution ESI-Q-TOF mass spectrometer was employed to identify and quantify cow's milk caseins using average mass information [8]; various glycosylated forms of kCNs (20939 Da, 19988 Da, 19040 Da, and 19005 Da), aS2CN (26074 Da), aS1CN (23615 Da) and bCN (23982 Da) were identified. Averaged molecular mass was also used to identify variants and PTMs of milk proteins from Danish Holstein

cows; thus were detected aS1CN-8P (23613 D), aS1CN-9P (23692 Da), aS2CN-11P (25226 Da), aS2CN-12P (25306 Da), aS2CN-13P (25386 Da), bCN B (24087 Da), bCN A1 (24019 Da), bCN A2 (23978 Da), kCN A (19035 Da), kCN B (19003 Da), and glycosylated forms of kCN (>19000 Da), bLG B (18280 Da), bLG A (18366 Da), and aLA (14184 Da) [9]. In a pooled milk sample from Jersey and Holstein-Friesian cows and using averaged masses, more variants were identified [10]: κ-CN A-1P (19035 Da), κ-CN E-1P (19004 Da), κ-CN B-1P (19002 Da), αs2-CN A-11P (25224 Da), αs2-CN A-12P (25304 Da), αs1-CN B-8P (23611 Da), αs1-CN C-8P (23539 Da), αs1-CN B-9P (23691 Da), αs1-CN C-9P (23619 Da), β-CN A1-5P (24020 Da), β-CN A2-5P (23980 Da), β-CN B-5P (24088 Da), β-CN F-5P (24033 Da), β-CNI-5P (23963 Da), α-LA B (14184 Da), β-LG A (18364 Da), β-LG B (18278 Da), and β-LG C (18287 Da). Very similar results were recently obtained using a LTQ linear ion-trap mass spectrometer to analyse major milk proteins and relate them to casein micelle sizes; average masses were assigned to aS1CN-8P (23611 Da), aS1CN-9P (23691 Da), aS2CN-11P (25225 Da), aS2CN-12P (25304 Da), bCN B (24088 Da), bCN A1 (24109 Da), bCN A2 (23983 Da), bCN I (23965 Da), kCN A (19034 Da), kCN B (19002 Da), kCN A with 2 GalNAc-Gal(NeuAC) (20932 Da), kCN B with 2 GalNAc-Gal(NeuAC) (20900 Da), kCN B with 1 GalNAc-Gal(NeuAC) (19983 Da), bLG B (18278 Da), bLG A (18363 Da), and aLA (14186 Da)[11]. By using a high resolution ESI-Q-TOF instrument, accurate monoisotopic masses of bCN variants A1, A2, B and C were obtained; these variants were quantified by extracting their chromatograms using specific multi-charged ions [12]. In a different study, direct infusion of intact cow milk proteins into a ESI-LTQ Orbitrap MS, whose high mass resolution gives access to accurate monoisotopic masses, also led to the identification of aS1CN-8P (23601.4350 Da) and bCN A1 (24009.2221 Da) and A2 (23968.1420 Da) variants [13].

2. Method details

2.1. Mobile phase gradients

Three gradients were tested. The 1st gradient (referred to 3-40% B hereafter) was as follows: starting conditions 3% B, held for 2.5 min, ramping to 40% B in 27.5 min, ramping to 99% B in 1 min and held at 99% B for 4 min, lowering to 3% B in 0.1 min, equilibration at 3% B for 4.9 min. The 2nd gradient (referred to 24-28-45% B hereafter) reproduced LC separation method from [14] with modifications: starting conditions 24.3% B held for 2.5 min, ramping to 28.8% B in 1 min, ramping to 45.2% B in 28.5 min, ramping to 99% B in 1.5 min and held for 1.5 min, lowering to 20% B in 0.1 min, equilibration at 20% B for 4.9 min. The 3rd gradient (referred to 20-28-40% B hereafter) was as follows: starting conditions 20% B, ramping to 28% B in 2.5 min, ramping to 40% B in 27.5 min, ramping to 99% B in 1 min and held for 4 min, lowering to 20% B in 0.1 min, equilibration at 20% B for 4.9 min.

2.2. Mobile phase flow rates

Three mobile phase flow rates were tested: 100, 200 and 300 μL/min.

2.3. Mobile phase compositions

Three sets of mobile phases were tested. In the 1st set, phase A contained acetonitrile (ACN) with 0.1% FA and phase B contained H_2O with 0.1% FA. In the 2nd set, phase A contained ACN with 0.1% FA and 0.02% trifluoroacetic acid (TFA), and phase B contained H_2O with 0.1% FA and 0.02% TFA. In the 3rd set, phase A contained ACN with 0.1% FA and 0.1% TFA, and phase B contained H_2O with 0.1% FA and 0.1% TFA.

2.4. Column temperature

The Aeris™ columns tested were stable when operated at up to 90°C. Three column temperatures were tested: 45°C, 60°C and 75°C.

2.5. Column stationary phases

Two HPLC separation columns were tested. One column was Aeris™ PEPTIDE XB-C18 (1.7 µm particle size, 200 Å pore size, 150 x 2.1 mm dimensions, C18 reverse phase core-shell silica from Phenomenex). The other column was Aeris™ WIDEPORE XB-C8 (3.6 µm particle size, 200 Å pore size, 150 x 2.1 mm dimensions, C8 reverse phase core-shell silica from Phenomenex). Being intended for different applications, these columns had the same dimensions (150 x 2.1 mm) but varied in their particle size. The C18 column has a 1.7 µm particle size and is designed for peptide separation; the C8 column has a 3.6 µm particle size and is designed for the separation of moderately hydrophobic proteins. These columns offer improved resolution and increased peak capacity for proteins and peptides as the uniform porous shell of the Aeris core-shell particle reduces the time it takes for proteins and peptides to adsorb/desorb to its surface resulting in decreased band broadening and high pore permeability for improved separation of very large proteins.

The efficiency (N) of the column was calculated using the following formula: $N=L/2d_P$, where L is the column length and d_P is the particle diameter. The interstitial volume (Vm) of the column was calculated using the following formula: $Vm=\Pi*r^2*L*W$, where r is the radius of the column, and W is the column percentage of interstitial porosity which can be approximated at 55% for core-shell columns [15]. Efficiency is measured by the number of theoretical plates (N), which is inversely proportional to particle size (dp) and directly proportional to column length (L). Conceptually, a plate refers to one complete equilibrated transfer (or partition) of a solute between the mobile and stationary phases. As particle size diminishes, efficiency increases, and more resolution is achieved. The same resolution can be achieved if the length of the column is reduced by the same factor as the particle size, thus shortening the analysis time. Column efficiency (N) and interstitial volumes (Vm) were computed; they differ significantly from one report to the other. Efficiency is measured by

the number of theoretical plates (N), which is inversely proportional to particle size (dp) and directly proportional to column length (L). Conceptually, a plate refers to one complete equilibrated transfer (or partition) of a solute between the mobile and stationary phases. As particle size diminishes, efficiency increases, and more resolution is achieved. The same resolution can be achieved if the length of the column is reduced by the same factor as the particle size, thus shortening the analysis time.

2.6. Integration of UV traces

Peak areas of UV chromatograms were integrated automatically along the retention time (RT) window of standards using the "Compounds - Chromatogram" function of DataAnalysis 4.2 SR2 software and the following parameters, S/N of 3, area threshold of 0%, intensity threshold of 3%, skim ratio of 0 and smoothing width of 3.

2.7. Mass scanning rates

Scanning was performed at two different spectra rates: 0.7 Hz, or 0.3 Hz (*i.e.* one scan every 1.5 or 3.0 seconds, respectively), with the mass range set at 600-3000 m/z. Individual data points of Base Peak Chromatogram (BPC) were visualised using Expressionist Refiner MS 9.1 software (Genedata).

2.8. Mass scanning ranges

MS optimisation tests included scanning across two different predefined acquisition windows, either 600–6000 m/z or 600-3000 m/z, with the scanning rate parameters set at 0.3 Hz (*i.e.* 3 sec/scan).

2.9. Linearity of calibration, sensitivity, LOD, LOQ, and working range of external standards

Standard curves were obtained for all standards. From the initial 10 mg/mL stock solutions, aCN, bCN, kCN, aLA, bLG and BSA standards were diluted down using 50% solution

A/50% MilliQ H_2O to the following concentrations: 7.50, 5.00, 2.50, 1.00, 0.75, 0.50, 0.25 and 0.10 mg/mL. Standard curves were run in duplicate. MS data files were visualised and further processed post-acquisition using DataAnalysis 4.2 SR2 software (Bruker DaltonikGmbh).

MS files were batch processed post-acquisition using QuantAnalysis software version 2.2 (Bruker DaltonikGmbh). Parameters were: smoothing using Gauss algorithm, 3 s width, and 7 cycles; peak detection with 1 S/N threshold, 0.1% relative area and intensity thresholds, 0.1 skim ratio, and 1 smoothing width; mass spectrum calculation using the actual width of peaks. Myoglobin spiked at a 0.2 mg/mL concentration was used as the internal standard and ratio automatically produced by QuantAnalysis software. Following automatic detection and quantitation, all peaks were manually validated. Output results were exported in Excel software (version 10, Microsoft). Finally, the summed responses were adjusted for purity levels of the individual standards. The adjusted responses were then plotted against the concentration range and trend lines obtained.

The sensitivity of the method was estimated using the slope of the calibration curves. A slope with a high positive value indicates that the method is highly sensitive. The more sensitive, the better a method is able to detect small variations in analyte concentration.

The limit of detection (LOD) for each standard was computed with the following formula: 3*(standard error/slope). The limit of quantitation (LOQ) for each standard was computed with the following formula: 10*(standard error/slope).

The working range was the interval between the LOQ and the upper concentration of the analyte in the samples tested in this study (10 mg/mL) for which linearity was demonstrated.

2.10. Internal standard (IS) calibration curve, matrix effect, and sensitivity of the instrument

Myoglobin was chosen as an IS because it should not be naturally present in bovine milk. Furthermore, its mass was distinctly different to that of the milk proteins of interest as well as displaying minimum coelution with the most abundant milk proteins. Matrix effect was assessed using a post-extraction spike method.

The myoglobin calibration curve was prepared by diluting a 10 mg/mL stock solution of horse myoglobin in either 50% Solution A/50% H_2O, Jersey milk sample or Holstein milk sample at the following concentrations: 7.50, 5.00, 2.50, 1.00, 0.75, 0.50, 0.25 and 0.10 mg/mL. IS curves were run in duplicate. MS data files were visualised and EICs produced using QuantAnalysis 2.2 software and the ion series indicated in Table A in S2 File. Peak area (PA) integration and plotting was performed as detailed above.

Matrix effect was computed by subtracting IS response in milk sample (either Jersey or Holstein samples) to that in Solution A and dividing the difference by the response in Solution A. Results were then converted to percent.

Thus the formula applied was: $(PA_{milk} – PA_{SolA}) / PA_{SolA} * 100$.

Sensitivity was assessed using the signal-to-noise ratio (S/N). Noise was obtained by averaging over the RT of myoglobin (16.5-18.5 min) the PAs of triplicated blank samples (50% Solution A/50% H_2O run under our optimum HPLC-MS conditions). This noise corresponded to the Limit of Blank (LOB)[16].

2.10. Reproducibility, precision, and selectivity of proteins in standards and milk samples

Standards and milk samples spiked with myoglobin IS were run three times using our optimum LC-MS conditions to assess the reproducibility of the HPLC separation. Unspiked standards and milk samples were also run in triplicate to assess the effect of the IS on the protein separation. MS data files were visualised and EICs produced using QuantAnalysis 2.2

software and the ion series indicated in Table A in S2 File. Peak area integration and plotting was performed as detailed above. Responses were plotted against the concentration range and trend lines obtained using Excel software.

Precision was evaluated across repeated measurement results and expressed by coefficient of variation (CV) of replicate results. Student t-tests were performed in Excel software (two-tailed, equal variance) for each protein of interest to test which proteins significantly differed between cow breeds.

Selectivity was verified using EICs of the proteins of interest (Table A in S2 File). If the response of a set of proteins can be distinguished from all other responses attributable to matrix components, then the method is demonstrated to be selective.

2.12. Deconvolution, mass accuracy and mass resolution

In DataAnalysis 4.2, the DISSECT algorithm (Bruker DaltonikGmbh) was also applied on representative MS data files in order to detect individual compounds over a retention time range. The following parameters were applied: S/N threshold of 4, 3 maximum overlapping compounds, and 0.1% cut-off intensity. The multiply charged ion spectra of "dissected" compounds were subsequently deconvoluted using Maximum Entropy algorithm with the following parameters: masses from 10000 to 70000, 0.1 data point spacing, 60000 resolving power, and normal resolution. Accurate mass assignment of isotopically resolved deconvoluted data was performed using the Sophisticated Numerical Annotation Procedure (SNAP) algorithm (Bruker Daltonik GmbH) and the following parameters were utilised: 0.9 quality factor threshold, 10 S/N threshold, 10% relative intensity threshold, and 30 maximum charge state. Observed deconvoluted masses from the standard samples were then manually recorded on a spreadsheet. As isotopically resolved spectra were obtained for all protein standards, monoisotopic mass assignments could be obtained. The exception was BSA, whose high molecular weight prevented isotopic resolution. As such, the average mass was

obtained for BSA. Information pertaining to the quality of mass spectra for each deconvoluted mass of protein variants was retrieved from DataAnalysis software. This included mass resolution, S/N, intensity and full width at half maximum (FWHM). TheGrand Average of Hydropathy (GRAVY) index was computed online (http://web.expasy.org/protparam/; [17]) using the manually curated AA sequence of the mature protein. The larger the GRAVY number, the more hydrophobic the protein is. Utilising the manually curated sequences, theoretical monoisotopic or average masses were computed using the online Peptide Mass Calculator tool from Peptide Protein Research Ltd. (http://www.peptidesynthetics.co.uk/tools/). Mass accuracy was assessed by subtracting the observed deconvoluted masses to the theoretical ones.

3. Supplementary Results

3.1. Elution order does not follow GRAVY index for proteins with PTMs

Table B in S2 File indicates the GRAVY index of the protein variants; the smaller the GRAVY value, the more hydrophobic the protein. GRAVY value is computed based on AA sequence information only, it does not take into account PTMs such as phosphorylation and glycosylation. Based on this index, proteins should elute as follows: aS2CN A-10P = aS2CN A-11P = aS2CN A-12P = aS2CN A-13P = aS2CN A-14P > aS1CN B-8P = aS1CN B-9P >kCN A-1P = kCN A-2P >kCN B-1P = kCN B-2P = kCN B-1P+G > BSA >aLA B = aLA B+G >Myo (IS) >bCN B-5P >bCN A1-5P >bCN A2-5P >bCN I-5P >bLG A >bLG B = bLG D. Based on RT, the obtained elution order of the standards utilising our optimum LC method is: kCN A-1P > aS2CN A-10P >kCN A-2P > aS2CN A-11P > aS2CN A-12P > aS2CN A-14P >kCN B-1P+G > aS2CN A-13P >kCN B-1P >kCN B-2P >aLA B+G > aS1CN B-8P >aLA B > BSA > aS1CN B-9P >Myo (IS) >bCN B-5P>bCN A1-5P>bLG B >bCN A2-5P >bLG A>bCN I-5P >bLG D. RTs are as expected from GRAVY predictions, with the exception of aS1CNs which are highly phosphorylated and contain 17.6% (35/199) basic

amino acids (R, K and H). Protein hydrophobicity should decrease with degree of phosphorylation, except if basic amino acids are positively charged, as would be the case under acidic LC-MS conditions, which would reduce the net charge of the protein and render it more hydrophobic [18]. The whey protein aLA bears no phosphorylation and, based on GRAVY index, is predicted to elute with BSA and myoglobin after kCNs and before bCNs, as observed in our study but conflicts with published results[9-11, 14, 19-20].

4. References:

1. Stelwagen K. Milk proteins. In: Fuquay JW, Fox PF, McSweeney PLH, editors. The Encyclopedia of Dairy Sciences. 2nd ed. Academic Press; 2011; pp 359-366.

2. Singh H. Functional properties of milk proteins. In: Fuquay JW, Fox PF, McSweeney PLH, editors. The Encyclopedia of Dairy Sciences. 2nd ed. Academic Press; 2011; pp 887-893.

3. Caroli AM, Chessa S, Erhardt GJ. Invited review: milk protein polymorphisms in cattle: effect on animal breeding and human nutrition. J Dairy Sci. 2009; 92(11): 5335-52.

4. Mann M, Meng CK, Fenn JB. Interpreting mass spectra of multiply charged ions. Anal. Chem. 1989; 61: 1702-1708.

5. Ayoub D, Jabs W, Resemann A, Evers W, Evans C, Main L, et al. Correct primary structure assessment and extensive glyco-profiling of cetuximab by a combination of intact, middle-up, middle-down and bottom-up ESI and MALDI mass spectrometry techniques. MAbs. 2013; 5(5): 699-710.

6. Chevalier F. Mass spectrometric methods. In: Fuquay JW, Fox PF, McSweeney PLH, editors. The Encyclopedia of Dairy Sciences. 2nd ed. Academic Press; 2011; pp 198-205.

7. Sargaeva N, Wilson J, Stacey C. LC/MS Study of Casein Proteins in Milk. PerkinELmer Application Note. 2014.

8. Bonizzi I, Buffoni JN, Feligini M. Quantification of bovine casein fractions by direct chromatographic analysis of milk. Approaching the application to a real production context. J Chromatogr A. 2009; 1216(1): 165-8.

9. Frederiksen PD, Andersen KK, Hammershøj M, Poulsen HD, Sørensen J, Bakman M, et al. Composition and effect of blending of noncoagulating, poorly coagulating, and well-coagulating bovine milk from individual Danish Holstein cows. J Dairy Sci. 2011; 4(10): 4787-99.

10. Jensen HB, Poulsen NA, Andersen KK, Hammershøj M, Poulsen HD, Larsen LB. Distinct composition of bovine milk from Jersey and Holstein-Friesian cows with good, poor,

or noncoagulation properties as reflected in protein genetic variants and isoforms. J Dairy Sci. 2012; 95(12): 6905-17.

11. Day L, Williams RP, Otter D, Augustin MA. Casein polymorphism heterogeneity influences casein micelle size in milk of individual cows. J Dairy Sci. 2015; 98(6): 3633-44.

12. Givens I, Aikman P, Gibson T, Brown R. Proportions of A1, A2, B and C β-casein protein variants in retail milk in the UK. Food Chem. 2013; 139(1-4): 549-52.

13. Pierri G, Kotoni D, Simone P, Villani C, Pepe G, Campiglia P, et al. Analysis of bovine milk caseins on organic monolithic columns: an integrated capillary liquid chromatography-high resolution mass spectrometry approach for the study of time-dependent casein degradation. J Chromatogr A. 2013; 1313: 259-69.

14. Bobe G, Beitz DC, Freeman AE, Lindberg GL. Separation and Quantification of Bovine Milk Proteins by Reversed-Phase High-Performance Liquid Chromatography. J Agric Food Chem. 1998; 46(2): 458-463.

15. Taylor T. HPLC column transfer from fully porous to core–shell particles in three simple equations. The Column. 2015; 11(9): 2-4.

16. Armbruster DA, Pry T. Limit of blank, limit of detection and limit of quantitation. ClinBiochem Rev. 2008; Suppl 1: S49-52.

17. Gasteiger E, Hoogland C, Gattiker A, Duvaud S, Wilkins MR, Appel RD, et al. Protein Identification and analysis tools on the ExPASy server. In: Walker JM, editor. The Proteomics Protocols Handbook. Humana Press; 2005; pp 571-607

18. Steen H, Jebanathirajah JA, Rush J, Morrice N, Kirschner MW. Phosphorylation analysis by mass spectrometry: myths, facts, and the consequences for qualitative and quantitative measurements. Mol Cell Proteomics. 2006; 5(1): 172-81.

19. Bordin G, CordeiroRaposo F, de la Calle B, Rodriguez AR. Identification and quantification of major bovine milk proteins by liquid chromatography. J Chromatogr A. 2001; 928(1): 63-76.

20. Bonfatti V, Grigoletto L, Cecchinato A, Gallo L, Carnier P. Validation of a new reversed-phase high-performance liquid chromatography method for separation and quantification of bovine milk protein genetic variants. J Chromatogr A. 2008; 1195(1-2): 101-6.
