# Supplementary material for: Quantitation and Identification of Intact Major Milk Proteins for High-Throughput LC-ESI-Q-TOF MS Analyses
Source: PLoS One. 2016 Oct 17;11(10):e0163471. doi: 10.1371/journal.pone.0163471 (PMC5066972; doi:10.1371/journal.pone.0163471)
Supplement: S2 File — (PDF) [file pone.0163471.s002.pdf]

Figure A . Optimisation of MS acquisition.

Vincent et al. 2016

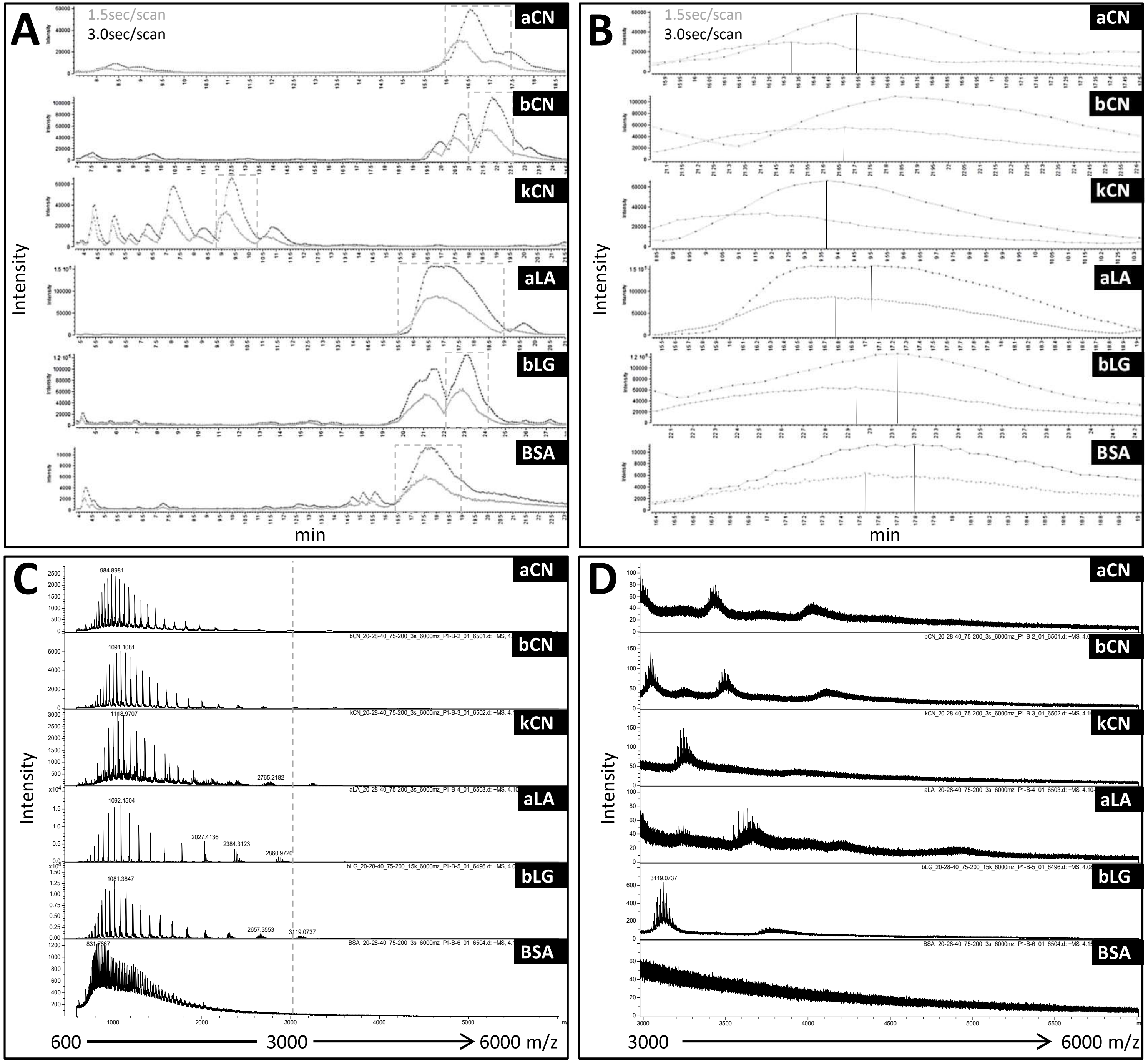

Scan rate (A-B) and mass range (C-D) were refined. BPCs on panel A are displayed for each standard from 5 to 24 min along the x axis; the most intense base peak per standard is boxed with a dotted line. This boxed area is zoomed in on panel B to further visualise individual data points. The whole BPCs displayed in panel A have been averaged across all peaks to produce the average spectra in panel C ranging from 600 to 6000 m/z; the dotted line marks 3000 m/z. The mass range 3000-6000 m/z is zoomed in on panel D to illustrate the lack of spectral peaks along this range.

Figure B. (1/3) Elution profile and spectral data for each protein of interest.

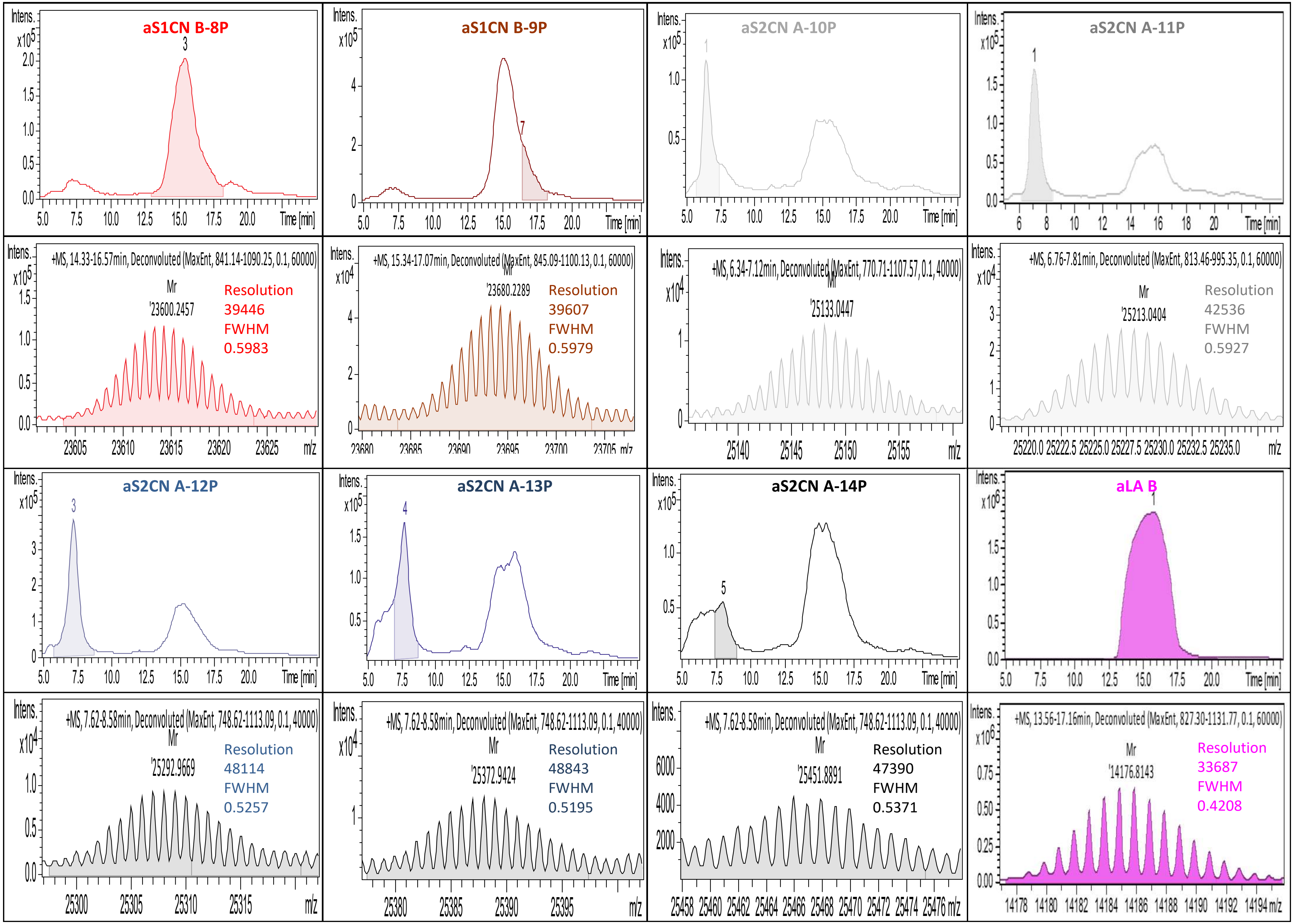

Figure B. (2/3) Elution profile and spectral data for each protein of interest.

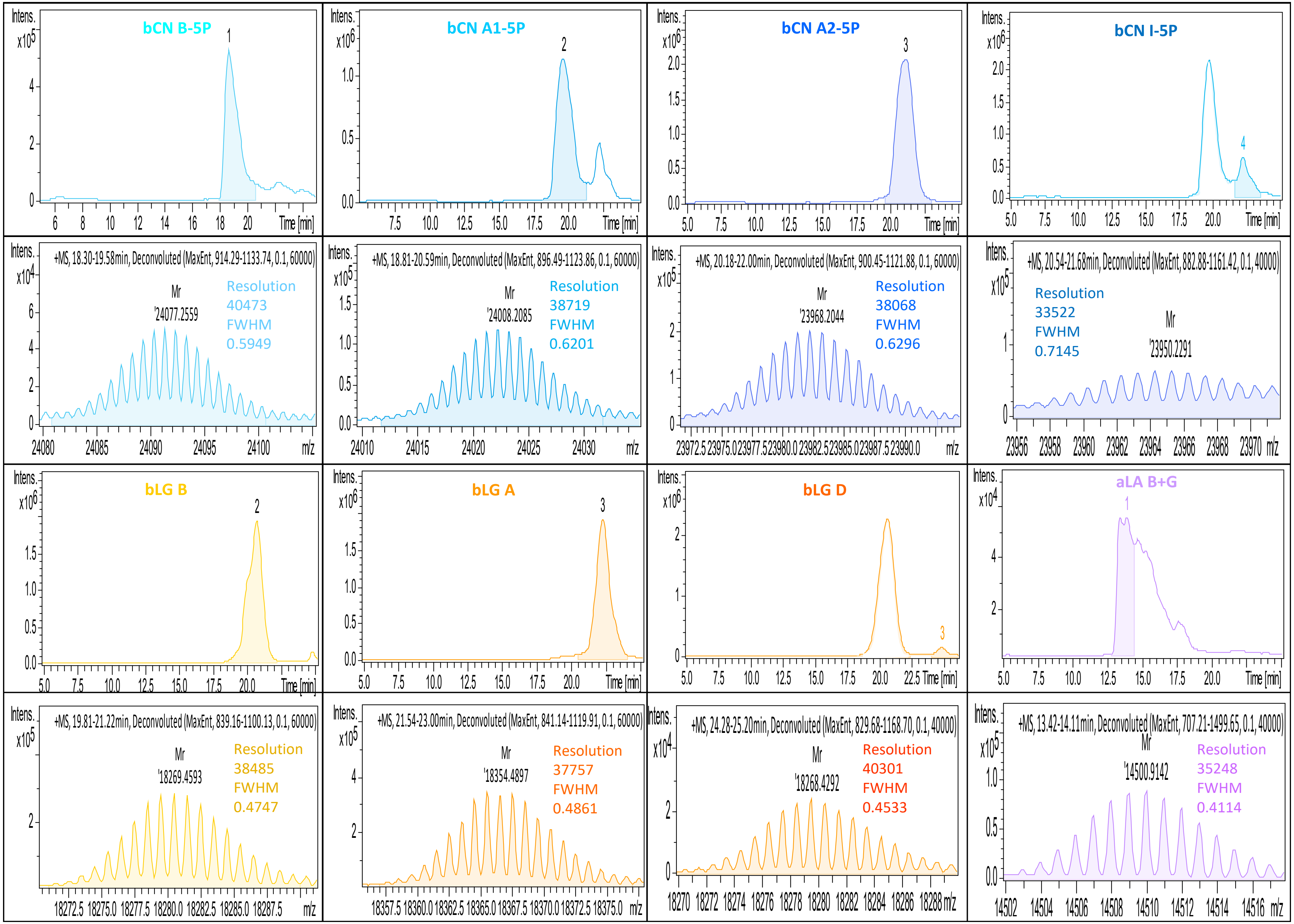

Figure B. (3/3) Elution profile and spectral data for each protein of interest.

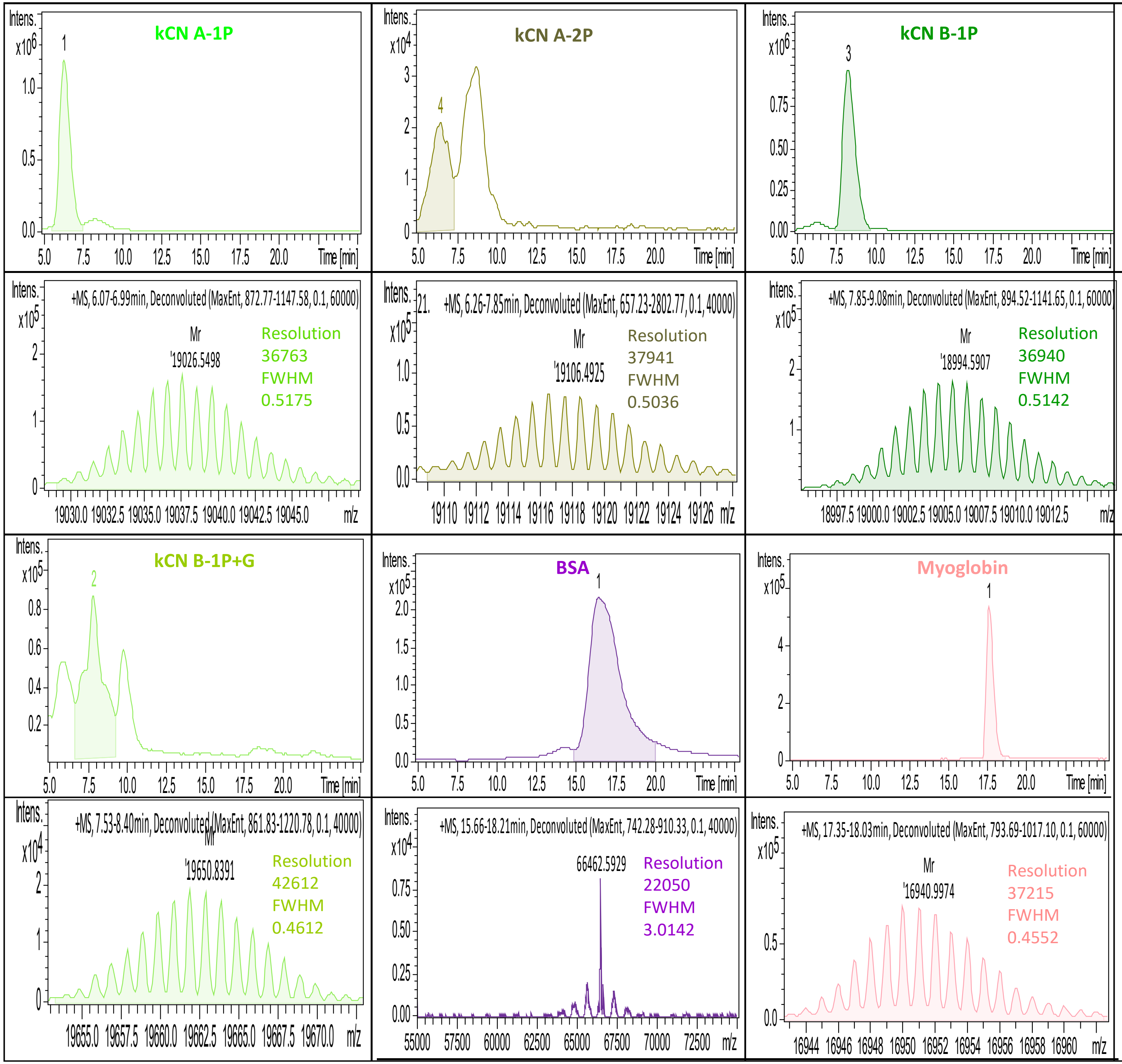

Top panels display EICs obtained using ion series from Table1; integrated peak area are shaded. Second panels from the top display multicharged ion spectra along 600-3000 m/z range. Third panels from the top display deconvoluted accurate mass using Maximum Entropy deconvolution algorithm; resolution and full width at half maximum (FWHM) are also indicated. Bottom panels display a zoomed in area centred on deconvoluted mass to illustrate high resolution of the spectra.

Figure C. Linearity of calibration of external standards.

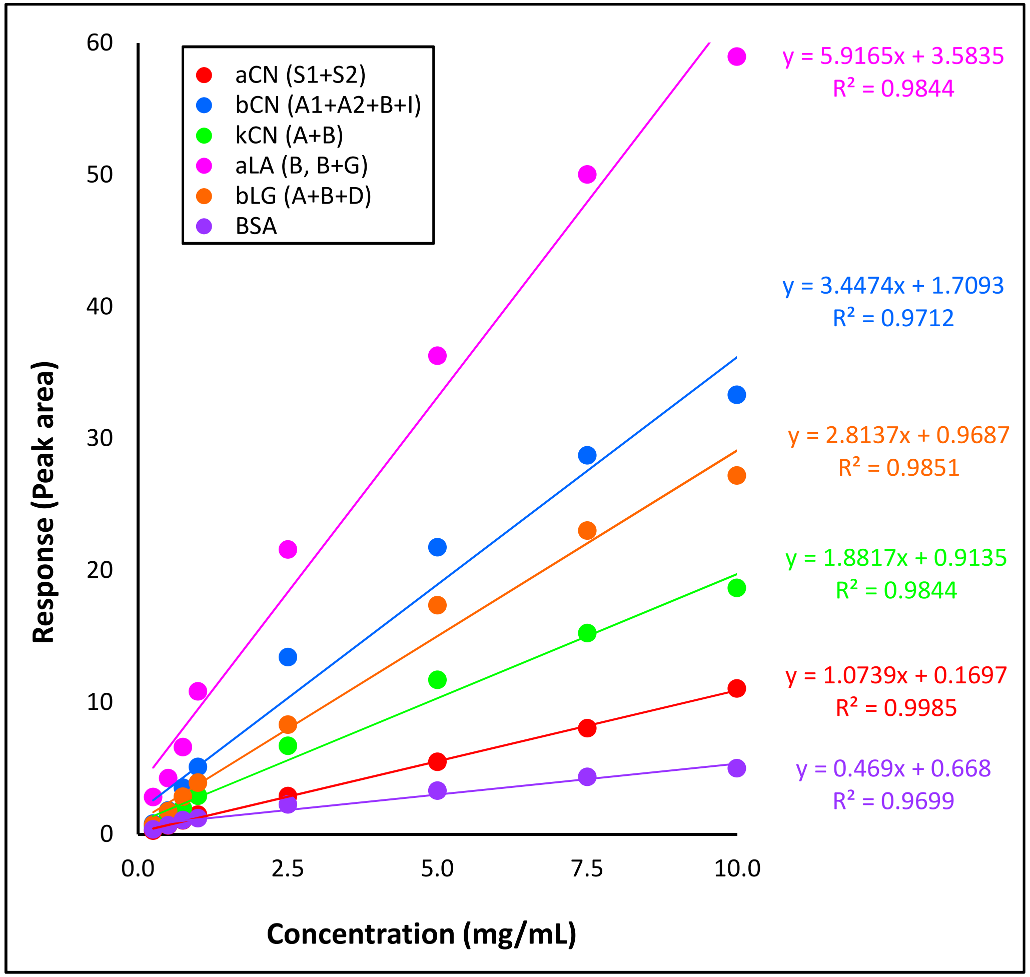

Two technical replicates were used to average the EIC peak areas. All external standards were solubilised in 50% Solution A. Trend lines and Pearson correlation coefficient (R2) values are displayed.

Figure D. Linearity of calibration of myoglobin internal standard and matrix effect.

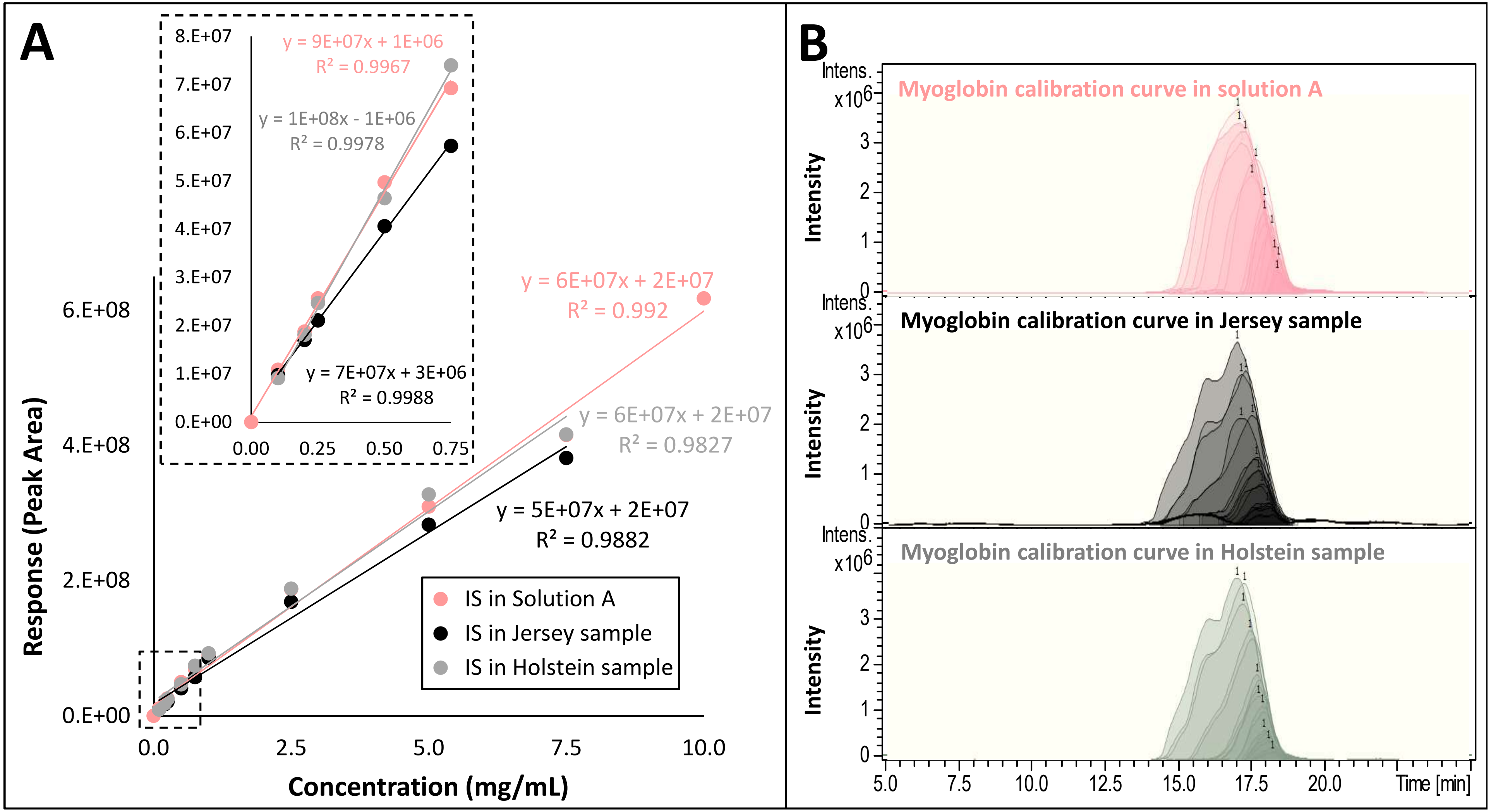

Two technical replicates were used to average the EIC peak areas. Three matrices were tested, 50% Solution A, Jersey milk sample, and Holstein milk sample. Panel A displays Myoglobin's response plotted against the whole concentration range (0-10 mg/mL). In the inset of panel A the concentration range has been limited to 0-1 mg/mL. Trend lines and Pearson correlation coefficients (R2) are displayed. Panel B displays the EICs of myoglobin prepared as increasing concentration (0.1-7.5mg/mL shown here) in each of the matrix along 5-25 min. The integrated peak areas are shaded.

Figure E. Reproducibility of protein external standard EICs across six replicates.

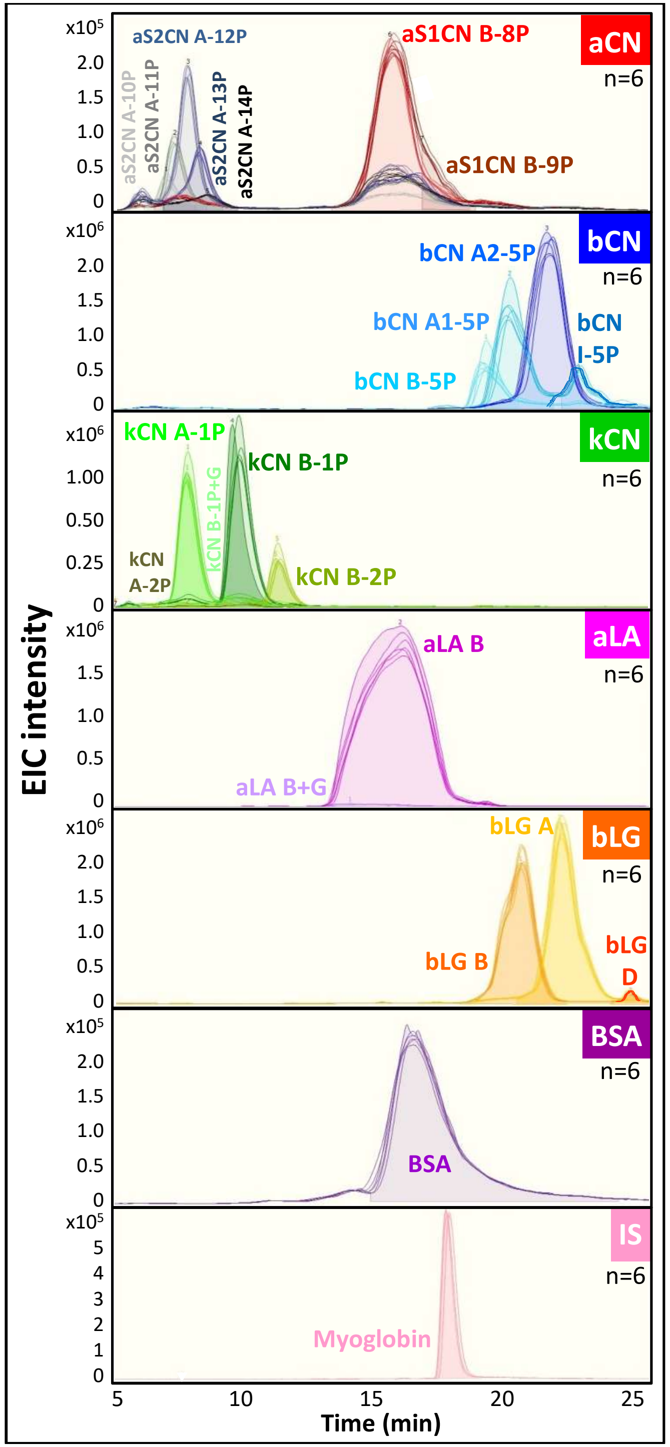

Three repeated injections on two different days, once spiked with myoglobin internal standard and once neat were used. The EIC of each protein of interest is color-coded and bear the abbreviated name of the variant. The shaded area corresponds to the peak area that was integrated as a proxy to protein quantity.

Figure F. Synopsis highlighting the benefit of using EICs over UV trace or BPC for protein quantitation.

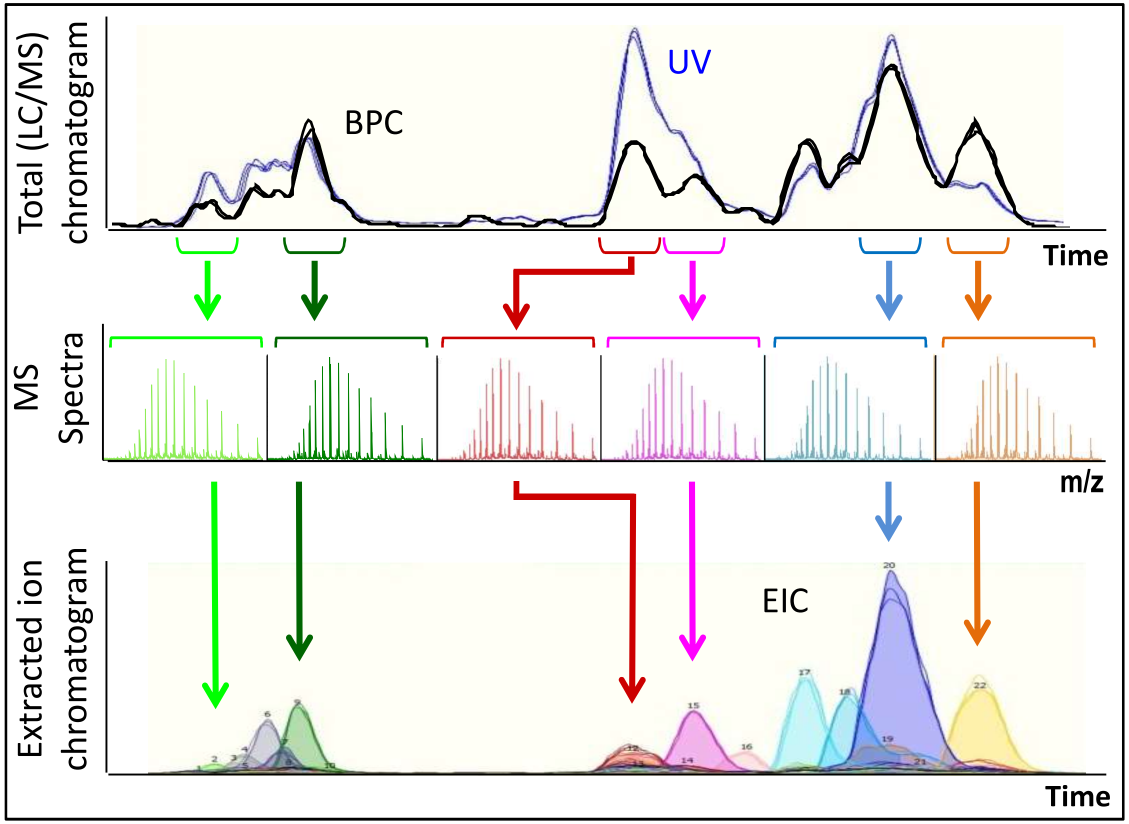

Proteins that partly co-elute following LC separation can only be accurately quantified by using given ions from their mass spectra to produce their EICs and integrating the EICs' area as a proxy to protein quantity. UV traces or BPCs both lack the resolution to distinguish between overlapping proteins.

Table A. List of cow milk protein variants quantified in this study.

Protein name	Protein code	RT apex (min)	Ion series (m/z) for Extracted Ion Chromatogram (EIC)
alpha S1 casein variant B with 8 phosphorylations	aS1CN B-8P	15.43	762.7257; 788.1166; 815.1211; 843.8743; 875.5735; 908.7102; 945.0179; 984.3516; 1027.1057; 1073.7012; 1124.8295; 1181.0196; 1243.1265; 1312.9660; 1389.2573; 1476.0230; 1574.3576; 1686.7394; 1816.4104
alpha S1 casein variant B with 9 phosphorylations	aS1CN B-9P	16.90	788.1168; 815.2588; 844.3741; 875.6098; 911.2101; 948.2142; 987.6421; 1030.5391; 1077.3829; 1128.5909; 1184.9695; 1247.2826; 1316.5764; 1393.9029; 1481.0211; 1579.6888; 1692.3803; 1822.5616
alpha S2 casein variant A with 10 phosphorylations	aS2CN A-10P	6.60	786.4078; 812.2047; 839.3109; 868.1826; 899.1893; 932.3799; 968.2408; 1058.6513; 1094.4032; 1144.1019; 1197.8118; 1257.6524
alpha S2 casein variant A with 11 phosphorylations	aS2CN A-11P	7.08	789.3213; 814.6872; 841.4438; 870.9386; 901.6017; 935.3413; 970.7007; 1009.5718; 1051.4684; 1097.1820; 1147.1024; 1201.6296; 1261.5587; 1327.9593; 1401.7330; 1484.0086
alpha S2 casein variant A with 12 phosphorylations	aS2CN A-12P	7.30	767.7852; 791.8517; 817.3621; 844.5748; 873.6986; 904.8648; 938.3774; 974.3548; 1013.3269; 1055.5079; 1101.3122; 1151.3256; 1206.1511; 1266.3075
alpha S2 casein variant A with 13 phosphorylations	aS2CN A-13P	8.15	747.2341; 770.3701; 794.3830; 820.0057; 847.3089; 876.4538; 907.6877; 941.3673; 977.4302; 1016.4469; 1058.1650; 1104.1287; 1154.9609; 1209.9599; 1269.5981
alpha S2 casein variant A with 14 phosphorylations	aS2CN A-14P	7.90	728.1969; 749.5850; 772.2694; 796.3716; 822.0287; 849.3963; 879.1786; 909.9961; 944.2659; 980.5045; 1019.3852; 1061.4954; 1108.2227; 1157.9041
alpha lactalbumin variant B	aLA B	15.80	710.3007; 747.5744; 789.0546; 835.4696; 887.5609; 946.7290; 1014.2109; 1092.1498; 1183.1611; 1290.5395; 1419.4929; 1577.2124; 1774.1134
alpha lactalbumin variant B glycosylated	aLA B+G	13.50	807.1165; 854.0036; 907.3157; 967.7360; 1036.7881; 1116.4636; 1209.4180; 1320.0940; 1451.0998; 1612.2210; 1813.6223
beta casein variant A1 with 5 phosphorylations	bCN A1-5P	19.67	775.9205; 801.7817; 829.3260; 858.9438; 890.7163; 924.9396; 961.8943; 1001.9334; 1045.4511; 1092.9267; 1144.9217; 1202.1127; 1265.3377; 1335.5740; 1502.4192; 1602.4707; 1716.8965; 1848.8826
beta casein variant A2 with 5 phosphorylations	bCN A2-5P	21.15	774.6299; 800.4153; 827.9816; 857.5153; 889.2381; 923.4010; 960.3287; 1000.2660; 1043.7116; 1091.1064; 1143.0184; 1200.1177; 1263.2334; 1333.3538; 1411.7368; 1499.9261; 1599.8547; 1714.0327; 1845.8191
beta casein variant B with 5 phosphorylations	bCN B-5P	18.76	804.0184; 831.7437; 861.4137; 893.2421; 927.6297; 964.6547; 1004.8101; 1048.4951; 1096.0719; 1148.1649; 1205.5725; 1269.0227; 1339.4159; 1418.1583; 1506.7784; 1607.1052; 1721.7413; 1854.2840
beta casein variant I with 5 phosphorylations	bCN I-5P	22.20	960.2969; 1000.2673; 1043.7132; 1091.1090; 1143.0658; 1200.1688; 1263.2300; 1333.4087; 1410.7296; 1498.7738; 1598.7585; 1712.8122
beta lactoglobulin variant A	bLG A	22.10	707.4514; 735.6717; 766.2697; 799.5516; 835.8495; 875.6037; 919.3457; 967.6503; 1021.3772; 1081.3731; 1148.9139; 1225.4408; 1312.9003; 1413.8150; 1531.5496; 1670.6903; 1837.6585; 2041.7292
beta lactoglobulin variant B	bLG B	20.65	704.0654; 732.1901; 762.6859; 795.7976; 831.9386; 871.5034; 915.0445; 963.1508; 1016.5708; 1076.3453; 1143.5365; 1219.7068; 1306.8210; 1407.1980; 1524.3810; 1662.8676; 1829.0536
beta lactoglobulin variant D	bLG D	24.70	704.1039; 732.2277; 762.6948; 795.8112; 831.3915; 871.5068; 915.0315; 963.1379; 1016.5893; 1076.3292; 1143.5365; 1219.7071; 1306.7549; 1407.1969; 1524.3792; 1662.8677
bovine serum albumin	BSA	16.51	747.7548; 756.2491; 764.9476; 773.8395; 782.9061; 792.2301; 801.7902; 811.5230; 821.5470; 831.8010; 842.3110; 853.1087; 864.1506; 875.5008; 899.1319; 911.4582; 924.1183; 937.1112; 950.4742
kappa casein variant A with 1 phosphorylation	kCN A-1P	6.44	794.2455; 828.6933; 866.3333; 907.5350; 952.8754; 1002.9616; 1058.6340; 1120.8439; 1190.8381; 1270.1627; 1360.7713; 1465.4370; 1587.4715; 1731.6931; 1904.8598
kappa casein variant A with 2 phosphorylations	kCN A-2P	6.83	865.7596; 910.8413; 952.3842; 1002.4571; 1062.4800; 1124.9194; 1195.1631; 1274.7737; 1365.7572; 1593.2160
kappa casein variant B with 1 phosphorylation	kCN B-1P	8.28	827.3404; 864.9012; 906.0195; 951.2667; 1001.3047; 1056.8574; 1118.9571; 1188.8232; 1268.0501; 1358.5464; 1462.9797; 1584.8062; 1728.7048; 1901.5674
kappa casein variant B with 2 phosphorylations	kCN B-2P	9.82	804.6199; 846.9331; 893.9743; 946.4931; 1005.5246; 1072.5561; 1149.0928; 1237.4840; 1340.5254; 1608.1322; 1787.0290
kappa casein variant B-1P glycosylated	kCN B-1P+G	7.90	787.0335; 819.7849; 855.3843; 894.2199; 937.2875; 984.1520; 1035.8427; 1093.3890
myoglobulin (internal standard)	Myo (IS)	17.54	707.3013; 737.9600; 771.5100; 808.1500; 848.5600; 893.1700; 942.7300; 998.1300; 1060.4500; 1131.0100; 1211.7900; 1304.9300; 1413.5930; 1542.0098

Indicated are proteins full name, their abbreviated code, the retention time (RT) at their apex as a 10 mg/mL solution standard, and the ion series used to produce their Extracted Ion Chromatogram (EICs) using a +/- 0.1 m/z tolerance.

Table B. Relevant publically available information on the cow milk protein variants quantified in this study. The table is split into two parts.

Protein code	UniProt number	Number of AA residues (mature form)	Maturation processing (SP, PP, other) [a]	Sequence variation (position in mature form)	PTMs (position in mature form) [b]	GRAVY [c]	Theoretical monoisotopic mass (Daltons)
aS1CN B-8P	P02662	214 (199)	SP 1-15	A(53), Q(59), E(192)	8xS* (46, 48, 64, 66, 67, 68, 75, 115) [+640D]	-0.704	23600.472
aS1CN B-9P	P02662	214 (199)	SP 1-15	sqce from aS1CN B-8P	9xS* (41, 46, 48, 64, 66, 67, 68, 75, 115) [+720D]	-0.704	23680.472
aS2CN A-10P	P02663	222 (207)	SP 1-15	E(33), A(47), T(130)	10xS* (8, 9, 10, 13, 31, 56, 57, 58, 61, 143) [+800D]	-0.918	25133.343
aS2CN A-11P	P02663	222 (207)	SP 1-15	E(33), A(47), T(130)	11xS* (8, 9, 10, 13, 31, 56, 57, 58, 61, 143, ?) [+880D]	-0.918	25213.343
aS2CN A-12P	P02663	222 (207)	SP 1-15	E(33), A(47), T(130)	12xS* (8, 9, 10, 13, 31, 56, 57, 58, 61, 143, ?, ?) [+960D]	-0.918	25293.343
aS2CN A-13P	P02663	222 (207)	SP 1-15	E(33), A(47), T(130)	13xS* (8, 9, 10, 13, 31, 56, 57, 58, 61, 143, ?, ?, ?) [+1040D]	-0.918	25373.343
aS2CN A-14P	P02663	222 (207)	SP 1-15	E(33), A(47), T(130)	14xS* (8, 9, 10, 13, 31, 56, 57, 58, 61, 143, ?,?,?,?) [+1120D]	-0.918	25453.343
aLA B	P00711	142 (123)	SP 1-19	R(10), D(?)		-0.453	14176.798
aLA B+G	P00711	142 (123)	SP 1-19	R(10), D(?)	2*Gal/Man (45) [+324D]	-0.453	14500.902
bCN A1-5P	P02666	224 (209)	SP 1-15	sqce from bCNA2-5P: P → H(67)	5xS* (15, 17, 18, 19, 35) [+400D]	-0.362	24008.317
bCN A2-5P	P02666	224 (209)	SP 1-15	R(25), E(36), E(37), P(67), Q(72), L(88), M(93), H(106), S(122), L/P (137/138), P(152), Q(?)	5xS* (15, 17, 18, 19, 35) [+400D]	-0.355	23968.311
bCN B-5P	P02666	224 (209)	SP 1-15	sqce from bCNA2-5P: P → H(67), S → R(122)	5xS* (15, 17, 18, 19, 35) [+400D]	-0.380	24077.386
bCN I-5P	P02666	224 (209)	SP 1-15	sqce from bCNA2-5P: M → L(93)	5xS* (15, 17, 18, 19, 35) [+400D]	-0.345	23950.355
bLG A	P02754	178 (162)	SP 1-16	sqce from bLGB: G → D(64), A → V(118)		-0.167	18355.446
bLG B	P02754	178 (162)	SP 1-16	E(45), P(50), I(56), Q(59), G(64), K(70), I(78), E(108), A(118), P(126), D(129), E(158)		-0.162	18269.409
bLG D	P02754	178 (162)	SP 1-16	sqce from bLGB: E → Q(45)		-0.162	18268.410
BSA	P02769	607 (583)	SP 1-18, PP 19-24	A → T (224)		-0.479	66462.966
kCN A-1P	P02668	190 (169)	SP 1-21	Q → PyrE(1), R(10), R(97), S(104), T(135), T(136), D(148), S(155)	1xS* (149) [+80D] + E*(1)[-17.026D]	-0.557	19026.542
kCN A-2P	P02668	190 (169)	SP 1-21	Q → PyrE(1), R(10), R(97), S(104), T(135), T(136), D(148), S(155)	2xS* (127, 149) [+160D] + E*(1)[-17.026D]	-0.557	19106.542
kCN B-1P	P02668	190 (169)	SP 1-21	sqce from kCNA-1P: Q → PyrE(1), T → I(136), D → A(148)	1xS* (149) [+80D] + 1xE*(1) [-17.026D]	-0.495	18994.589
kCN B-2P	P02668	190 (169)	SP 1-21	sqce from kCNB-1P	2xS* (127, 149) [+160D] + 1xE*(1) [-17.026D]	-0.495	19074.589
kCN B-1P+G	P02668	190 (169)	SP 1-21	sqce from kCNB-1P	1*GalNAc-Gal(NeuAC) (?) [+656D]	-0.495	19650.817
Myo (IS)	P68082	154 (153)	initial M(1) removed			-0.396	16940.956

Indicated are their Uniprot accession number, the number of amino acid residues in both the preprocessed and the mature forms, the processed domains, the sequence allelic variations and their position in the sequence of the mature form, the number of post-translational modifications and their position in the sequence of the mature form, the manually curated amino acid sequence, the grand average of hydropathicity (GRAVY) index, and the theoretical mass. [a] processing as part of protein maturation events include removal of the Signal Peptide (SP), and the Propeptide (PP), or the removal of the initial Methionine (M) residue; [b] post-translational modifications(PTMS) are coded as follows: phosphoserine residues (S*), N-terminus pyroglutamate (E*). Their position in the mature form of the protein are indicated in round brackets () and the mass shifts resulting from it are indicated in square brackets []. Question marks (?) indicates that the position of the PTM is not known; [c] the grand average of hydropathicity (GRAVY) index was computed online (web.expasy.org/protparam/) using the AA sequence manually curated of the mature protein.

Table B. Second part: amino acid sequences.

Protein code	AA sequence retrieved from UniProt and modified based on Farrell et al 2004 [c]
aS1CN B-8P	(MKLLILTCLVAVALA)RPKHPIKHQGLPQEVLNENLLRFFVAPFPEVFGKEKVNELSKDIGS*ES*TEDQAMEDIKQMEAES*IS*S*S*EEIVPNS*VEQKHIQKEDVPSERYLGYLEQLLRLKKYKVPQLEIVPNS*AEERLHSMKEGIHAQQKEPMIGVNQELAYFYPELFRQFYQLDAYPSGAWYYVPLGTQYTDAPSFSDIPNPIGSENSEKTTMPLW
aS1CN B-9P	(MKLLILTCLVAVALA)RPKHPIKHQGLPQEVLNENLLRFFVAPFPEVFGKEKVNELS*KDIGS*ES*TEDQAMEDIKQMEAES*IS*S*S*EEIVPNS*VEQKHIQKEDVPSERYLGYLEQLLRLKKYKVPQLEIVPNS*AEERLHSMKEGIHAQQKEPMIGVNQELAYFYPELFRQFYQLDAYPSGAWYYVPLGTQYTDAPSFSDIPNPIGSENSEKTTMPLW
aS2CN A-10P	(MKFFIFTCLLAVALA)KNTMEHVS*S*S*EESIIS*QETYKQEKNMAINPS*KENLCSTFCKEVVRNANEEEYSIGS*S*S*EES*AEVATEEVKITVDDKHYQKALNEINQFYQKFPQYLQYLYQGPIVLNPWDQVKRNAVPITPTLNREQLSTSEENSKKTVDMES*TEVFTKKTKLTEEEKNRLNFLKKISQRYQKFALPQYLKTVYQHQKAMKPWIQPKTKVIPYVRYL
aS2CN A-11P	(MKFFIFTCLLAVALA)KNTMEHVS*S*S*EESIIS*QETYKQEKNMAINPS*KENLCSTFCKEVVRNANEEEYSIGS*S*S*EES*AEVATEEVKITVDDKHYQKALNEINQFYQKFPQYLQYLYQGPIVLNPWDQVKRNAVPITPTLNREQLSTSEENSKKTVDMES*TEVFTKKTKLTEEEKNRLNFLKKIS*QRYQKFALPQYLKTVYQHQKAMKPWIQPKTKVIPYVRYL
aS2CN A-12P	(MKFFIFTCLLAVALA)KNTMEHVS*S*S*EESIIS*QETYKQEKNMAINPS*KENLCSTFCKEVVRNANEEEYSIGS*S*S*EES*AEVATEEVKITVDDKHYQKALNEINQFYQKFPQYLQYLYQGPIVLNPWDQVKRNAVPITPTLNREQLSTSEENS*KKTVDMES*TEVFTKKTKLTEEEKNRLNFLKKIS*QRYQKFALPQYLKTVYQHQKAMKPWIQPKTKVIPYVRYL
aS2CN A-13P	(MKFFIFTCLLAVALA)KNTMEHVS*S*S*EESIIS*QETYKQEKNMAINPS*KENLCSTFCKEVVRNANEEEYSIGS*S*S*EES*AEVATEEVKITVDDKHYQKALNEINQFYQKFPQYLQYLYQGPIVLNPWDQVKRNAVPITPTLNREQLSTS*EENS*KKTVDMES*TEVFTKKTKLTEEEKNRLNFLKKIS*QRYQKFALPQYLKTVYQHQKAMKPWIQPKTKVIPYVRYL
aS2CN A-14P	(MKFFIFTCLLAVALA)KNTMEHVS*S*S*EESIIS*QETYKQEKNMAINPS*KENLCS*TFCKEVVRNANEEEYSIGS*S*S*EES*AEVATEEVKITVDDKHYQKALNEINQFYQKFPQYLQYLYQGPIVLNPWDQVKRNAVPITPTLNREQLS*TSEENS*KKTVDMES*TEVFTKKTKLTEEEKNRLNFLKKIS*QRYQKFALPQYLKTVYQHQKAMKPWIQPKTKVIPYVRYL
aLA B	(MMSFVSLLLVGILFHATQA)EQLTKCEVFRELKDLKGYGGVSLPEWVCTTFHTSGYDTQAIVQNNDSTEYGLFQINNKIWCKDDQNPHSSNICNISCDKFLDDDLTDDIMCVKKILDKVGINYWLAHKALCSEKLDQWLCEKL
aLA B+G	(MMSFVSLLLVGILFHATQA)EQLTKCEVFRELKDLKGYGGVSLPEWVCTTFHTSGYDTQAIVQNNDSTEYGLFQINNKIWCKDDQNPHSSNICNISCDKFLDDDLTDDIMCVKKILDKVGINYWLAHKALCSEKLDQWLCEKL
bCN A1-5P	(MKVLILACLVALALA)RELEELNVPGEIVES*LS*S*S*EESITRINKKIEKFQS*EEQQQTEDELQDKIHPFAQTQSLVYPFPGPI**H**NSLPQNIPPLTQTPVVVPPFLQPEVMGVSKVKEAMAPKHKEMPFPKYPVEPFTESQSLTLTDVENLHLPLPLLQSWMHQPHQPLPPTVMFPPQSVLSLSQSKVLPVPQKAVPYPQRDMPIQAFLLYQEPVLGPVRGPFPIIV
bCN A2-5P	(MKVLILACLVALALA)RELEELNVPGEIVES*LS*S*S*EESITRINKKIEKFQS*EEQQQTEDELQDKIHPFAQTQSLVYPFPGPIPNSLPQNIPPLTQTPVVVPPFLQPEVMGVSKVKEAMAPKHKEMPFPKYPVEPFTESQSLTLTDVENLHLPLPLLQSWMHQPHQPLPPTVMFPPQSVLSLSQSKVLPVPQKAVPYPQRDMPIQAFLLYQEPVLGPVRGPFPIIV
bCN B-5P	(MKVLILACLVALALA)RELEELNVPGEIVES*LS*S*S*EESITRINKKIEKFQS*EEQQQTEDELQDKIHPFAQTQSLVYPFPGPI**H**NSLPQNIPPLTQTPVVVPPFLQPEVMGVSKVKEAMAPKHKEMPFPKYPVEPFTE**R**QSLTLTDVENLHLPLPLLQSWMHQPHQPLPPTVMFPPQSVLSLSQSKVLPVPQKAVPYPQRDMPIQAFLLYQEPVLGPVRGPFPIIV
bCN I-5P	(MKVLILACLVALALA)RELEELNVPGEIVES*LS*S*S*EESITRINKKIEKFQS*EEQQQTEDELQDKIHPFAQTQSLVYPFPGPIPNSLPQNIPPLTQTPVVVPPFLQPEV**L**GVSKVKEAMAPKHKEMPFPKYPVEPFTESQSLTLTDVENLHLPLPLLQSWMHQPHQPLPPTVMFPPQSVLSLSQSKVLPVPQKAVPYPQRDMPIQAFLLYQEPVLGPVRGPFPIIV
bLG A	(MKCLLLALALTCGAQA)LIVTQTMKGLDIQKVAGTWYSLAMAASDISLLDAQSAPLRVYVEELKPTPEGDLEILLQKWEN**D**ECAQKKIIAEKTKIPAVFKIDALNENKVLVLDTDYKKYLLFCMENSAEPEQSL**V**CQCLVRTPEVDDEALEKFDKALKALPMHIRLSFNPTQLEEQCHI
bLG B	(MKCLLLALALTCGAQA)LIVTQTMKGLDIQKVAGTWYSLAMAASDISLLDAQSAPLRVYVEELKPTPEGDLEILLQKWENGECAQKKIIAEKTKIPAVFKIDALNENKVLVLDTDYKKYLLFCMENSAEPEQSLACQCLVRTPEVDDEALEKFDKALKALPMHIRLSFNPTQLEEQCHI
bLG D	(MKCLLLALALTCGAQA)LIVTQTMKGLDIQKVAGTWYSLAMAASDISLLDAQSAPLRVYVE**Q**LKPTPEGDLEILLQKWENGECAQKKIIAEKTKIPAVFKIDALNENKVLVLDTDYKKYLLFCMENSAEPEQSLACQCLVRTPEVDDEALEKFDKALKALPMHIRLSFNPTQLEEQCHI
BSA	(MKWVTFISLLLLFSSAYS)(RGVFRR)DTHKSEIAHRFKDLGEEHFKGLVLIAFSQYLQQCPFDEHVKLVNELTEFAKTCVADESHAGCEKSLHTLFGDELCKVASLRETYGDMADCCEKQEPERNECFLSHKDDSPDLPKLKPDPNTLCDEFKADEKKFWGKYLYEIARRHPYFYAPELLYYANKYNGVFQECCQAEDKGACLLPKIETMREKVLASSARQRLRC**T**SIQKFGERALKAWSVARLSQKFPKAEFVEVTKLVTDLTKVHKECCHGDLLECADDRADLAKYICDNQDTISSKLKECCDKPLLEKSHCIAEVEKDAIPENLPPLTADFAEDKDVCKNYQEAKDAFLGSFLYEYSRRHPEYAVSVLLRLAKEYEATLEECCAKDDPHACYSTVFDKLKHLVDEPQNLIKQNCDQFEKLGEYGFQNALIVRYTRKVPQVSTPTLVEVSRSLGKVGTRCCTKPESERMPCTEDYLSLILNRLCVLHEKTPVSEKVTKCCTESLVNRRPCFSALTPDETYVPKAFDEKLFTFHADICTLPDTEKQIKKQTALVELLKHKPKATEEQLKTVMENFVAFVDKCCAADDKEACFAVEGPKLVVSTQTALA
kCN A-1P	(MMKSFFLVVTILALTLPFLGA)**E'**EQNQEQPIRCEKDERFFSDKIAKYIPIQYVLSRYPSYGLNYYQQKPVALINNQFLPYPYYAKPAAVRSPAQILQWQVLSNTVPAKSCQAQPTTMARHPHPHLSFMAIPPKKNQDKTEIPTINTIASGEPTSTPTTEAVESTVATLEDS*PEVIESPPEINTVQVTSTAV
kCN A-2P	(MMKSFFLVVTILALTLPFLGA)**E'**EQNQEQPIRCEKDERFFSDKIAKYIPIQYVLSRYPSYGLNYYQQKPVALINNQFLPYPYYAKPAAVRSPAQILQWQVLSNTVPAKSCQAQPTTMARHPHPHLSFMAIPPKKNQDKTEIPTINTIASGEPTSTPTTEAVESTVATLEDS*PEVIESPPEINTVQVTSTAV
kCN B-1P	(MMKSFFLVVTILALTLPFLGA)**E'**EQNQEQPIRCEKDERFFSDKIAKYIPIQYVLSRYPSYGLNYYQQKPVALINNQFLPYPYYAKPAAVRSPAQILQWQVLSNTVPAKSCQAQPTTMARHPHPHLSFMAIPPKKNQDKTEIPTINTIAS*GEPTSTPT**I**EAVESTVATLE**A**S*PEVIESPPEINTVQVTSTAV
kCN B-2P	(MMKSFFLVVTILALTLPFLGA)**E'**EQNQEQPIRCEKDERFFSDKIAKYIPIQYVLSRYPSYGLNYYQQKPVALINNQFLPYPYYAKPAAVRSPAQILQWQVLSNTVPAKSCQAQPTTMARHPHPHLSFMAIPPKKNQDKTEIPTINTIAS*GEPTSTPT**I**EAVESTVATLE**A**S*PEVIESPPEINTVQVTSTAV
kCN B-1P+G	(MMKSFFLVVTILALTLPFLGA)**E'**EQNQEQPIRCEKDERFFSDKIAKYIPIQYVLSRYPSYGLNYYQQKPVALINNQFLPYPYYAKPAAVRSPAQILQWQVLSNTVPAKSCQAQPTTMARHPHPHLSFMAIPPKKNQDKTEIPTINTIASGEPTSTPT**I**EAVESTVATLE**A**S*PEVIESPPEINTVQVTSTAV
Myo (IS)	(M)GLSDGEWQQVLNVWGKVEADIAGHGQEVLIRLFTGHPETLEKFDKFKHLKTEAEMKASEDLKKHGTVVLTALGGILKKKGHHEAELKPLAQSHATKHKIPIKYLEFISDAIIHVLHSKHPGDFGADAQGAMTKALELFRNDIAAKYKELGFQG

[d] AA sequence retreived from UniProt website (http://www.uniprot.org/) and further manually modifed as per Farrell et al. 2004, Holland et al 2004, and Bijl et al., 2014, to match variant sequence. AA residues removed as part of the processing are indicated in brackets (Signal peptide SP, propeptides PP, initial Methionine M(1)) (they are not considered to compute the theoretical monoisotopic mass). Phosphoresidues are indicated with an asterix. AA substitutions are underlined. Glycosylation sites are in grey.

Table C. Comparison of published method parameters for the analysis of major bovine milk proteins and our optimum method.

first author	year	milk type	protein extraction buffer	column supplier	stationary phase	column porosity	diameter (mm)	radius (mm) (r)	length (mm) (L)	particle size (um) (dp)	pore diameter (nm) (p)	injection volume (uL)	flow rate (mL/min) (F)	temperature (°C)	phase B composition	gradient ramp (%B)	gradient time (min)	MS type	Vm	N
Bijl	2014	skim milk	n.i.	BioRad	DEAE-5PW	n.i.	n.i.	n.i.	n.i.	n.i.	n.i.	25	0.4	40	n.i.	n.i.	33	none		
Bobe	1998	full cream	0.1M BisTris /6M GdnHCl /5.37mM Na citrate /19.5 mM DTT	Rainin Instruments	Microsorb MV C18	fully porous	4.6	2.30	250	5.0	30	20	1.20	RT	90%ACN /9.9%H2O /0.1%TFA	27-45	43	none	2825	25.0
Bonfatti	2008	full cream	Bobe's method	Agilent Technologies	Zorbax C8	fully porous	4.6	2.30	150	3.5	30	5	0.50	45	99.9%ACN /0.1%TFA	33-45	36	none	1695	21.4
Bonizzi	2009	skim milk	8M urea /165mM Tris /44mM Na citrate /0.3% 2ME	Phenomenex	Jupiter C4	fully porous	4.6	2.30	250	5.0	30	20	0.80	RT	99.9%ACN /0.1%TFA	30-50	40	ESI-Q-TOF	2825	25.0
Bordin	2001	skim milk	Bobe's method	Vydac	C4	fully porous	2.1	1.05	150	5.0	30	20	0.25	40	90%ACN /9.9%H2O /0.1%TFA	36.5-43.3	56	none	353	15.0
Day	2015	skim milk	Bobe's method	BioRad	Hi-Pore RP-318 C18	fully porous	4.6	2.30	250	5.0	30	n.i.	n.i.	n.i.	99.9%ACN /0.1%TFA	25-50	40	ESI-LTQ	2825	25.0
Frederiksen	2011	skim milk	Bobe's method	Phenomenex	Jupiter C4	fully porous	2.0	1.00	250	5.0	30	2	1.00	35	99.9%ACN /0.1%TFA	31-45	40	ESI-MS	534	25.0
Givens	2013	semi-skim	Bonizzi's method	Hichrom	ACE 5 C18	fully porous	2.1	1.05	150	5.0	30	5	0.20	45	99.99%ACN /0.01% TFA	33-43	44	ESI-Q-TOF	353	15.0
Jensen	2012	skim milk	Bobe's method	Phenomenex	Jupiter C4	fully porous	2.0	1.00	250	5.0	30	2	1.00	35	99.9%ACN /0.1%TFA	31-45	40	ESI-MS	534	25.0
Poulsen	2016	skim milk	Bobe's method	Phenomenex	Jupiter C4	fully porous	2.0	1.00	250	5.0	30	2	1.00	40	99.9%ACN /0.1%TFA	31-45	40	ESI-MS	534	25.0
Ramirez-Palomino	2014	full cream	Bonizzi's method	Phenomenex	Onyx C18	monolithic	4.6	2.30	100	2.0	13	10	3.00	40	95%ACN /4.9%H2O /0.1%TFA	2.5-10	5	none	914	25.0
Sargeava	2014	n.i.	Bonizzi's method	Phenomenex	Widepore XP C8	core-shell	2.0	1.00	100	3.6	20	2	0.40	50	99.9%ACN /0.1%FA /0.01%TFA	25-43	6	ESI-TOF	173	13.9
Vincent	2016	skim milk	Bobe's method	Phenomenex	Widepore XP C8	core-shell	2.1	1.05	150	3.6	20	3	0.20	75	99.9%ACN /0.1%FA /0.02%TFA	28-40	27.5	ESI-Q-TOF	286	20.8
Vincent	2016	skim milk	Bobe's method	Phenomenex	Widepore XP C8	core-shell	2.1	1.05	150	1.7	20	3	0.20	75	99.9%ACN /0.1%FA /0.02%TFA	28-40	27.5	ESI-Q-TOF	286	44.1

n.i., not indicated; Vm, column interstitial volume calculated as $Vm=\Pi*r^2*L*W$, where r is the column radius, L is the column length, and W=0.55 for monolithic/core-shell column, W=0.68 for fully porous column; N, column efficiency calculated as $N=L/2d_p$.
